# CRISPR/Cas9 in soybean research: a measured leap toward genetic refinement

**DOI:** 10.3389/fgeed.2026.1821910

**Published:** 2026-09-17

**Authors:** Smrutirekha Sahu, Pandian Rajendran, Sanket Arunrao Ghumde, Diksha Prakash, Shikha Dixit, Deepak Pawar, Dharavath Nagaraju, Vinutha T, Sandeep B. Adavi, Palaiyur Nanjappan Sivalingam, Ashish Marathe, Pramod Kumar Rai

**Affiliations:** 1 ICAR - National Institute of Biotic Stress Management, Raipur, Chhattisgarh, India; 2 Department of Soil and Crop Sciences, Texas A&M University, College Station, TX, United States; 3 ICAR – Directorate of Weed Research, Jabalpur, Madhya Pradesh, India; 4 Department of Genetics and Plant Breeding, College of Agriculture, Indira Gandhi Krishi Vishwavidyalaya, Raipur, Chhattisgarh, India; 5 ICAR – Indian Agricultural Research Institute, New Delhi, India

**Keywords:** base editing, biotic and abiotic stress, CRISPR/Cas9, genome editing, regulatory framework

## Abstract

Soybean [*Glycine max* (Linn.) Merr] is a globally important source of plant-based protein and edible oil, widely cultivated in India as a major oilseed crop. However, conventional breeding approaches for soybean are laborious, constrained by the genomic complexity of soybean, and face persistent challenges in genetic transformation. Clustered regularly interspaced short palindromic repeats (CRISPR)/Cas-based genome editing has emerged as a precise and efficient tool for rapid trait improvement in soybean. While many existing reviews of soybean have been published, this literature review addresses some fundamental challenges with respect to genome editing. This study summarizes recent advancements in soybean genome editing, offering insights into the practical informatics of soybean-specific genomic databases that are required for successful construct design. It also discusses emerging technologies such as base editing, prime editing, and the increasingly important role of artificial intelligence in accelerating target identification and construct design. It highlights applications in nutritional enhancement, abiotic and biotic stress tolerance, and improvement of key agronomic traits. Beyond the conventional topics, it addresses the current regulatory landscape that researchers must navigate for releasing genome-edited varieties and provides a comprehensive perspective on practical solutions to overcome major constraints in soybean genetic engineering.

## Graphical Abstract

**Figure:**
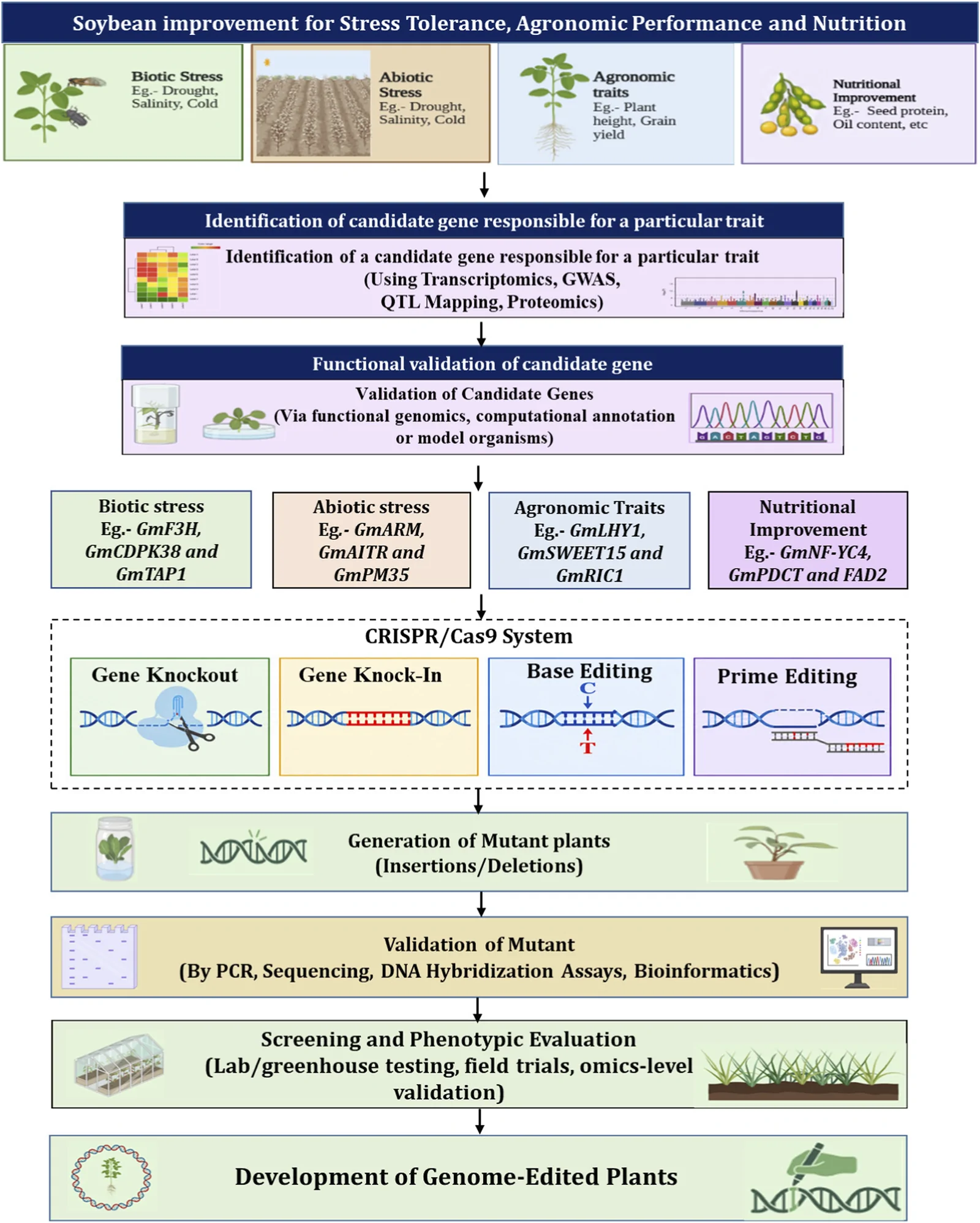
Made in BioRender Free trial period. https://app.biorender.com/illustrations/66cf6cabf6df30a07fce25a8.

## 1 Introduction

Oilseeds constitute a major component of Indian agriculture and are central to the economic and nutritional security of the country. Among the nine oilseed crops cultivated nationally, seven are sources of edible oils: soybean, groundnut, rapeseed-mustard, sunflower, sesame, safflower, and niger, while the remaining two, castor and linseed, are used for non-edible industrial purposes. Soybean (*Glycine max*) is native to East Asia and belongs to the family of *Fabaceae*, a subfamily of *Papilionoidea*. It has emerged as the most prominent legume crop globally, with annual production approaching 400 million tons (Monfort et al., 2025). Its agronomic significance is reinforced by its exceptional capacity for biological nitrogen fixation, contributing approximately 16 million tons of the 21 million tons of nitrogen fixed annually by seed legumes worldwide (Herridge et al., 2008).

Soybean is highly valued for its nutritional composition, consisting of roughly 40% protein and 20% oil. Approximately 85% of global soybean production is directed toward animal feed, while 7% is consumed directly by humans, and the remaining 8% is used for industrial applications and biofuel production (Voora et al., 2020). Brazil ranks as the largest producer, followed by, the United States, Argentina, and China, with India occupying fifth position in global soybean production (Fang et al., 2023). Within India, soybean cultivation is strategically organized across five agro-climatic zones. Production is predominantly in states such as Madhya Pradesh, Maharashtra, Rajasthan, Karnataka, Andhra Pradesh, and Chhattisgarh (Agarwal et al., 2013).

Despite a steady increase in demand, the genetic base of soybean has remained relatively narrow owing to domestication bottlenecks and reliance on conventional breeding approaches, necessitating the adoption of advanced molecular strategies to accelerate trait development (Nguyen et al., 2021). Among the genome editing platforms developed to date, including zinc-finger nucleases (ZFNs) and transcription activator-like effector nucleases (TALENs), the clustered regularly interspaced short palindromic repeats (CRISPR)/CRISPR-associated protein (Cas) has emerged as the most widely adopted third-generation genome editing technology (Nakamori, 2023; Bhuyan et al., 2023). CRISPR/Cas9 employs easily programmable guide RNAs to direct site-specific cleavage of genomic DNA. It is derived from an adaptive prokaryotic immune mechanism, unlike its predecessors, which require elaborate protein engineering and dimerization of *Fok I* nuclease domains (Osakabe and Osakabe, 2015; Wu et al., 2014). To address the canonical 5′-NGG-3′ requirement of SpCas9 that restricts targeting to GC-rich genomic regions, engineered variants including xCas9, Cas9-NG, SpG, and the near-PAMless SpRY have been developed with broadened target range (Ren et al., 2021). Another popular alternative is Cas12a (Cpf1), which recognizes T-rich protospacer adjacent motif (PAM) sequences and generates staggered DNA breaks. It demonstrated particular utility in soybean genome editing when deployed via DNA-free ribonucleoprotein delivery (Zetsche et al., 2015; Bao et al., 2020; Kim and Choi, 2021).

Soybean presents a uniquely complex editing landscape due to its paleopolyploid genome, in which approximately 75% of genes exist as multiple copies, and many agronomically relevant traits are under polygenic regulation (Yıldırım et al., 2023). CRISPR/Cas9 has been successfully deployed to improve soybean tolerance to abiotic stresses, including drought, salinity, and heavy metal accumulation (Shi et al., 2017; Xu et al., 2015), and to enhance resistance against diverse biotic stresses (Ali et al., 2015; Wang et al., 2016; Zhang Y. et al., 2022). The capacity to simultaneously target paralogous gene copies using single or multiplexed guide RNAs and using codon-optimized Cas9 variants tailored for soybean represents a critical advantage of this system for soybean improvement, although off-target mutations and regeneration efficiency remain notable technical challenges (Wu et al., 2014; Gupta et al., 2023; Michno et al., 2015).

This review comprehensively examines recent advances in CRISPR-based genome editing in soybean, with emphasis on system variants, trait improvement applications, existing technical limitations, relevant genomic databases, and the regulatory frameworks governing genome-edited crops in India and globally.

## 2 Types of CRISPR/Cas systems and mechanism of action

The CRISPR/Cas system was originally identified as an adaptive immune mechanism in prokaryotes, in which bacteria and archaea defend themselves against viruses by integrating fragments of viral DNA as spacer sequences. These sequences are later transcribed into crRNAs, which, together with Cas proteins, enable precise recognition and cleavage of invading nucleic acids (Barrangou, 2015). In soybean genome editing, the most widely applied nucleases are Cas9 and Cas12a due to their simple single-effector domain and high editing efficiency. Cas9, a type II nuclease, requires both crRNA and tracrRNA to function, whereas the type V-A nuclease Cas12a operates with only a crRNA, making them preferred tools for targeted gene modification in soybean (Koonin et al., 2017).

### 2.1 CRISPR/Cas9

Cas9 was originally discovered in bacteria, where it functions as a defense mechanism against viral attack. The most fundamental feature of CRISPR/Cas9 is an RNA-guided DNA recognition mechanism, which confers extraordinary programmability upon the Cas9 endonuclease. Cas9 is directed to a specific genomic locus by a chimeric single-guide RNA, a synthetic fusion of the CRISPR RNA (crRNA) and trans-activating crRNA (tracrRNA). The 20-nucleotide spacer sequence base pairs with the complementary strand of the target DNA through Watson–Crick base pairing (Freitas-Alves et al., 2025). The recognition of the target region requires a conserved protospacer adjacent motif (PAM) flanking the protospacer sequence, which corresponds to the trinucleotide 5′-*NGG-*3′ in the case of most extensively utilized *Streptococcus pyogenes.* The ability to redirect the nuclease to any genomic region simply by redesigning the sgRNA marked a paradigm shift from the comparatively laborious protein engineering requirements of ZFNs and TALENs. However, a key limitation associated with Cas9 deployment is the potential for off-target cleavage effects. It subsequently raises legitimate concerns regarding unintended alterations to genomic integrity and consequent phenotypic repercussions in the edited organism.

The functional versatility of CRISPR/Cas9 has been considerably enhanced through the identification and characterization of Cas9 orthologs derived from diverse bacterial species, including SaCas9 from *Staphylococcus aureus*, StCas9 from *Streptococcus thermophilus*, FnCas9 from *Francisella novicida*, CjCas9 from *Campylobacter jejuni*, and NmCas9 from *Neisseria meningitidis,* each recognizing distinct PAM sequences and thereby expanding the range of targetable genomic sites (Ran et al., 2015; Müller et al., 2016; Hirano et al., 2016; Kim E. et al., 2017; Hou et al., 2013). In the context of soybean improvement, CRISPR/Cas9 has emerged as the most widely used genome editing platform for numerous trait improvement applications such as soybean mosaic virus resistance (Zhang et al., 2020), enhanced insect resistance (Zhang Z. et al., 2022), improved salinity resistance (Wang et al., 2021), drought tolerance (Zhong et al., 2022), modification of leaf architecture (Cheng et al., 2019), regulation of floral development (Cheng et al., 2023), elevation of mono unsaturated fatty acid (MUFA) content (Li et al., 2023), reduction of beany flavor (Wang et al., 2020), and enhancement of seed protein content (Shen et al., 2022). These studies collectively underscored the transformative potential of this technology in soybean molecular breeding.

### 2.2 dCas9

The CRISPR/Cas9 system has been successfully repurposed as a dCas9-based platform for controlling how genes are switched on and off without making any permanent changes to the DNA sequence itself (Qi et al., 2013). While the mutant dCas9 retains the full sgRNA-directed DNA binding capacity of wild-type Cas9, it lacks endonuclease activity due to a point mutation (H840A) in its HNH catalytic domain. This strategy enables precise genome editing without inducing double-stranded breaks. The catalytically inert scaffold can also be exploited as a programmable platform for epigenetic modulation and cellular imaging (Karlson et al., 2021). Gene activation requires the design of sgRNA targeting −400 bp to −50 bp upstream of the translation start site (TSS), while transcriptional repression is achieved by targeting the −50 bp to +300 bp window encompassing the TSS. The functional specificity of this system is attributed to modular effectors that provide tunable and reversible control of gene regulatory networks without introducing permanent DNA modifications to the underlying DNA sequence.

Depending on the nature of the effector, the dCas9 platform operates either as a transcriptional repressor (CRISPRi) (Adli, 2018; Piatek et al., 2015) or activator (CRISPRa) (Casas-Mollano et al., 2020). Early implementations of CRISPRa employed fusions of dCas9 with VP64, a tetrameric repeat of the herpes simplex virus VP16 transactivation domain, to achieve targeted upregulation of endogenous genes at defined genomic loci (Pickar-Oliver and Gersbach, 2019). Similarly, the plant-specific SRDX domain has emerged as the most extensively validated transcriptional repressor in the plant CRISPRi systems, where it functions by recruiting corepressors and associated histone deacetylase complexes to silence transcription at target loci, with functional validation demonstrated primarily in *Arabidopsis* (Lowder et al., 2015). Freitas-Alves et al. (2024) demonstrated the functional application of CRISPR/dCas9 constructs fused with the transcriptional activator VP16/64 and a modified guide RNA 2.0 scaffold. They employed this construct to upregulate *GmExpA/GmExpA2* and *GmExpLB* genes in response to *Meloidogyne incognita* infection and observed a measurable reduction in gall numbers in the edited soybean lines. While many reports are not yet available in soybean, the clear potential of dCas9-based transcriptional tools for targeted improvement in soybean makes the experimental validation of CRISPRa and CRISPRi effector systems a strategic priority in soybean genome engineering research.

### 2.3 Cas12

Cas12 nucleases belong to the Class 2, Type V CRISPR/Cas systems, and possess unique structural and mechanistic properties that contribute to their versatility and specificity as genome editing tools. Amongst the two emerging effector complexes for plant systems, Cas12a (also known as *Cpf1*: CRISPR from *Prevotella and Francisella 1*) and Cas12f (also called Cas14) have received considerable research attention. The structures of the recognition lobe (REC) and nuclease (NUC) lobe in Cas12a are similar to those of Cas9. However, a key mechanistic distinction lies in the fact that Cas12a relies exclusively on its RuvC nuclease domain for cleaving both strands of target DNA, whereas Cas9 employs both RuvC and HNH domains in a coordinated dual-strand cleavage mechanism (Paul and Montoya, 2020). A unique structural element is the wedge (WED) domain, which stabilizes the crRNA-DNA hybrid duplex and facilitates productive interaction with the target region. Unlike SpCas9, which requires 5′-NGG-3′, Cas12a recognizes T-rich 5′-TTTN-3′ PAM sequences, substantially expanding the repertoire of targetable genomic sites across both bacterial and eukaryotic systems. Upon target binding, Cas12a undergoes conformational rearrangements within its RuvC domain that generate staggered double-strand breaks with short 5′ overhangs, in contrast to the blunt-ended cuts characteristic of Cas9 cleavage (Safari et al., 2020).

In soybean research, the application of CRISPR/Cas12a has expanded steadily across diverse trait improvement objectives and demonstrated its utility in modifying fatty acid profiles (Wang et al., 2025a), reducing accumulation of allergens (Liu et al., 2025), and enabling visual detection of *Phytophthora sojae* infection through integration with recombinase polymerase amplification (RPA) in a diagnostic biosensing platform (Guo et al., 2023a). A novel Cas12-SF01-based cytosine base editor that conferred herbicide resistance by targeting *GmALS1* and *GmALS3*, genes encoding acetolactate synthase isoforms, was developed. This system introduced precise C-to-T transitions at specific codons within each locus, expanding the practical utility of Cas12-based editing systems in soybean (Niu et al., 2024).

### 2.4 Cas14

Cas14 is a miniaturized variant within the Class 2, Type V CRISPR/Cas systems. It is distinguished by its compact size of 400–700 amino acids compared to the typically larger 1,200–1,500 amino acid structure of Cas12 proteins. It exclusively targets single-stranded DNA (ssDNA) without any requirement for a PAM sequence, thus enabling broader and more flexible targeting capabilities. This is in contrast to Cas12a, which requires a PAM sequence and targets dsDNA (Harrington et al., 2018). Structurally, Cas14 further deviates from Cas9 through the absence of the HNH nuclease domain, retaining only the RuvC-like domain for catalytic activity, which underlies its capacity to cleave ssDNA but not dsDNA under physiological conditions. Cas14 is hypothesized to have evolved primarily to confer bacterial immunity against ssDNA phages, and this restricted favor to ssDNA substrates correspondingly limits its genome editing utility in plant systems (Harrington et al., 2018). Although the application of this system has not been documented in soybean to date, its unique biochemical properties present several unexplored opportunities worthy of future investigation. The presence of ssDNA cleavage activity and miniature structure of Cas14 position it as a promising candidate for the development of a highly sensitive fluorometric biosensor capable of detecting soybean allergens or pathogen-derived nucleic acids with exceptional sensitivity and specificity, representing an avenue of applied research in future soybean breeding programs (Zhou et al., 2023).

### 2.5 Cas13

Cas13 enzymes represent a functionally distinct category within the CRISPR toolkit. They are classified under the Class 2, Type VI CRISPR effectors, which are distinguished by their ability to target and cleave RNA rather than DNA. The bilobed conformation of the structure consists of a REC lobe and a NUC lobe. The REC lobe performs functions reminiscent of other Class 2 CRISPR effectors, mediating the binding and stabilization of crRNA at the target site, while the NUC lobe houses two highly conserved higher eukaryote and prokaryote nucleotide-binding (HEPN) domains that assemble to form a catalytic ribonuclease activity site. After target binding, Cas13 cleaves the complementary ssRNA (single-stranded RNA) in *cis* and additionally degrades non-target ssRNA in *trans* through collateral cleavage activity (Abudayyeh et al., 2016; East-Seletsky et al., 2016). The Cas13 family is further subdivided into four subtypes: Cas13a, Cas13b, Cas13c, and Cas13d. Each of them differs considerably in their domain organization and crRNA structural configurations (Kordyś et al., 2022).

The activity of Cas13 has been strategically harnessed in the Specific High Sensitivity Enzymatic Reporter UnLOCKing (SHERLOCK) diagnostic platform. This enabled nucleic acid detection sensitively with minimized off-target RNA degradation concerns. Cas13 has been successfully employed in distinguishing genetically modified glyphosate-resistant soybean from wild-type varieties and holds considerable promise for detecting viral pathogens such as soybean mosaic virus (Abudayyeh et al., 2019). CRISPR/CasRX contains the compact RfxCas13d effector, which has been further engineered to simultaneously target positive and negative sense strands of the soybean mosaic virus HC-Pro gene, conferring robust resistance to soybean mosaic virus in the edited lines (Gao et al., 2024).

Collectively, the diverse array of CRISPR-associated nucleases illustrates the remarkable and rapidly expanding versatility of CRISPR-based genome engineering platforms. These systems differ fundamentally in their target substrate, PAM requirement, cleavage mechanism, and effector size, yet collectively, they represent a progressive continuum of technological innovation that has transformed plant genome engineering from a single tool to a multifaceted molecular toolkit. These platforms have been deployed across an impressive range of applications in soybean, as mentioned above, which underscores their transformative potential for accelerating soybean breeding programs. The structural and functional distinctions among these Cas systems are further illustrated in Figure 1 and summarized comparatively in Table 1.

**FIGURE 1:**
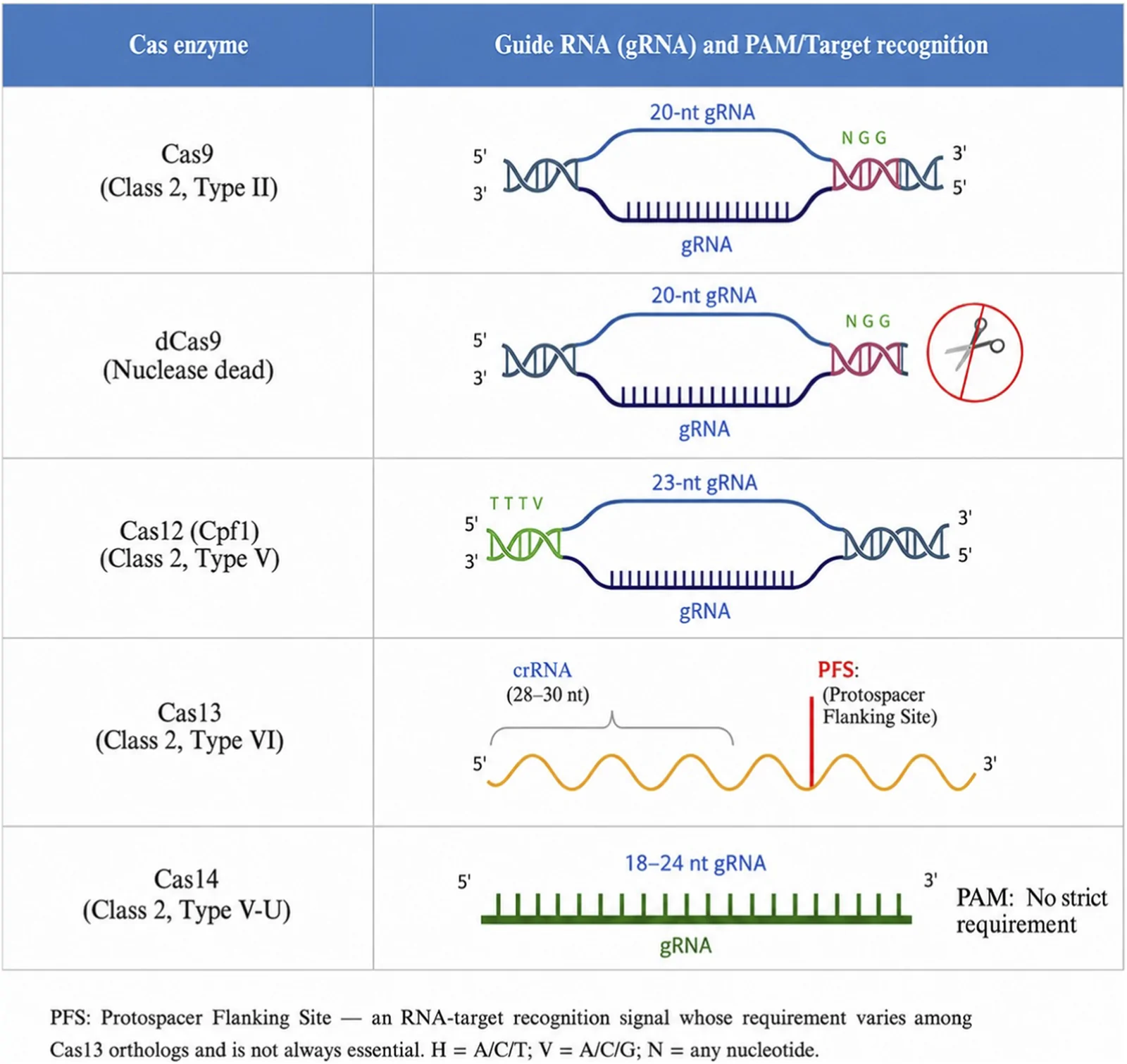
Comparative overview of CRISPR-Cas variants (Cas9, dCas9, Cas12, Cas13, and Cas14) illustrating their distinct guide RNA architectures, PAM/PFS requirements, and target substrates (dsDNA, ssRNA, and ssDNA). Created in BioRender. Rajendran (2026) https://BioRender.com/p6r9ze0.

**TABLE 1:** CRISPR-Cas-based systems and their specific features.

Features	Cas9	dCas9	Cas12a	Cas14	Cas13
Type/Class	Type II CRISPR/Cas	Catalytically inactive Cas9 mutant	Type V-A CRISPR/Cas	Type V-F CRISPR-Cas	Type VI CRISPR/Cas
Size of protein	∼1,368 amino acids (SpCas9)	Same as Cas9	∼1,300–1,500 amino acids	∼400–700 amino acids	∼900–1,200 amino acids
Type of enzyme	Dual nuclease domains (HNH +RuvC)	Catalytically inactive Cas9 variant	Single RuvC-like domain	Single RuvC-like domain	RNA-targeting nuclease (HEPN)
Nucleic acid target	dsDNA	Binds dsDNA, no cleavage	dsDNA	ssDNA	ssRNA
PAM requirement	Yes	Same as Cas9	Yes	No	No (but requires protospacer flanking site (PFS))
Cleavage pattern	Blunt-ended, double-strand breaks causing indels	No cleavage; binds DNA	Staggered double-strand breaks with 5′ overhang	Cleavage only on ssDNA	Cleavage only on ssRNA (Knockdown)
Guide RNA	dsDNA target + sgRNA (crRNA + tracrRNA)	Same as Cas9	dsDNA target + crRNA	ssDNA + crRNA	ssRNA target + crRNA
Applications	Gene disruption, knockouts	Regulation of gene expression, epigenetic modulation	Knockout	ssDNA detection	RNA knockdown, transcriptome editing
Limitations	Potential off-targets	Limited to binding-based applications	Collateral cleavage	Limited editing applications currently	Probable off-target RNA degradation
Sequencing analysis	Indel detection through Sanger sequencing	ChIP-based assays, imaging	Indel detection through NGS	Diagnostic assays	RNA sequencing or qPCR assays
Efficiency	High NHEJ	Highly adaptable for regulation	High HDR	Moderate edits	Rapid knockdown of viral RNA
Scalability	Moderate (2–8 gRNAs)	High	Excellent	High	Moderate

## 3 Soybean databases and genome editing pipeline

The reliability of CRISPR-based systems is fundamentally dependent on the precise recognition of target DNA or RNA sequences by guide RNAs, which makes obtaining accurately annotated genomic information for effective experimental design indispensable (Ran et al., 2013). These contemporary genomic databases provide detailed annotations of coding exons, sequence data, untranslated regions, introns, promoters, and gene variants that collectively enable the design of target-specific gRNAs. Given that CRISPR effectors may carry distinct PAM requirements and target-site constraints, database resources that facilitate effector-specific PAM site identification should be given more emphasis to broaden the practical scope of these editing platforms (Harrington et al., 2018).

The Soybase Genome Browser is powered by JBrowse and provides an interactive, highly customizable interface for exploring key soybean genome assemblies in Wm82.A2 and Wm82.a4.v1. It offers customizable data tracks for genes and transcripts, coordinate-based search across all 20 linkage groups, and seamless integration with analytical tools such as BLAST, germplasm panels, and SoyCyC metabolic pathway databases for functional and breeding applications (Brown et al., 2021).

Phytozome is another excellent resource that is maintained by the DOE Joint Genome Institute. It hosts the Williams 82 reference genome with comprehensive gene model annotations and robust comparative genomics relevant to guide RNA target identification (Goodstein et al., 2012). The Legume Information System (LIS) functions as an important comparative genomics platform integrating genetic, genomic, and trait data across numerous domesticated legumes, with soybean as a central reference species. It employs the ZZBrowse genome viewer as its interactive interface for exploring soybean genome assemblies and annotations in a cross-species context (Dash et al., 2016). Another reliable backup tool is the NCBI Genome database, which is a comprehensive, general-purpose repository hosting *Glycine max* and *Glycine soja* genome assemblies, offering direct access to NCBI BLAST for sequence homology searches and ClinVar for variant analysis (Sayers et al., 2021). Similarly, ENSEMBL Plants hosts *Glycine max* and *Glycine soja* genome assemblies with sophisticated comparative tools, browser-based visualization, gene tree construction, ortholog identification, and cross-database integration with UniProt, InterPro, and RefSeq annotations, collectively supporting functional characterization (Harrison et al., 2024). For soybean researchers focused on CRISPR/Cas-based genome editing, the combined utilization of SoyBase and Phytozome/ENSEMBL represents the current gold standard for target-site identification, guide RNA design, and functional genomic characterization. A curated summary of the key soybean genomic databases relevant for CRISPR-based gRNA design, including their primary features and accessibility, is listed in Table 2.

**TABLE 2:** Major soybean sequence and assembly databases.

Database name	Feature	Website	Accessed on:	Publicly available
SoyBase	Genome browser, gene annotations	https://soybase.org/	19/05/2026	Yes
Phytozome	Annotated soybean genomes	https://phytozome-next.jgi.doe.gov/	19/05/2026	Yes
Legume information system (LIS)	Soybean genome sequences, genome browser	https://legumeinfo.org/	19/05/2026	Yes
NCBI genome database	Genome repository	https://www.ncbi.nlm.nih.gov/genome/	19/05/2026	Yes
ENSEMBL plants	Gene models, comparative annotation	https://plants.ensembl.org/Glycine_max/Info/Index	19/05/2026	Yes

### 3.1 Genome editing pipeline in soybean, limitations, and corrective modifications

Genome editing in soybean follows a multi-step pipeline that integrates target identification, construct design, delivery, and validation of the guide RNA cassettes (Figure 2). The genomic databases serve as centralized hubs that enable users to access annotated plant gene families, examine genomic information of soybean genes, and retrieve related sequences and gene alignments that are essential for precise experimental design (Goodstein et al., 2012). The construct design is a critical determinant of genome editing success, where the target-site mutagenesis efficiency is influenced by several factors, including the choice of promoters driving Cas and gRNA expression, the quality of gRNA design, the number of gRNAs for single-guide RNA-based or multiplexed trait modification, and the selection of an expression vector for plant transformation (Freitas-Alves et al., 2025). In this regard, soybean-specific small nuclear RNA gene promoters have been shown to have mutation efficiency at 20.2%, highlighting their preference for achieving reliably edited plants (Sun et al., 2015). In addition, codon-optimized SpCas9, when driven by a strong constitutive promoter, showed an editing efficiency of 59.2% in transformed soybean plants (Bai et al., 2020).

**FIGURE 2:**
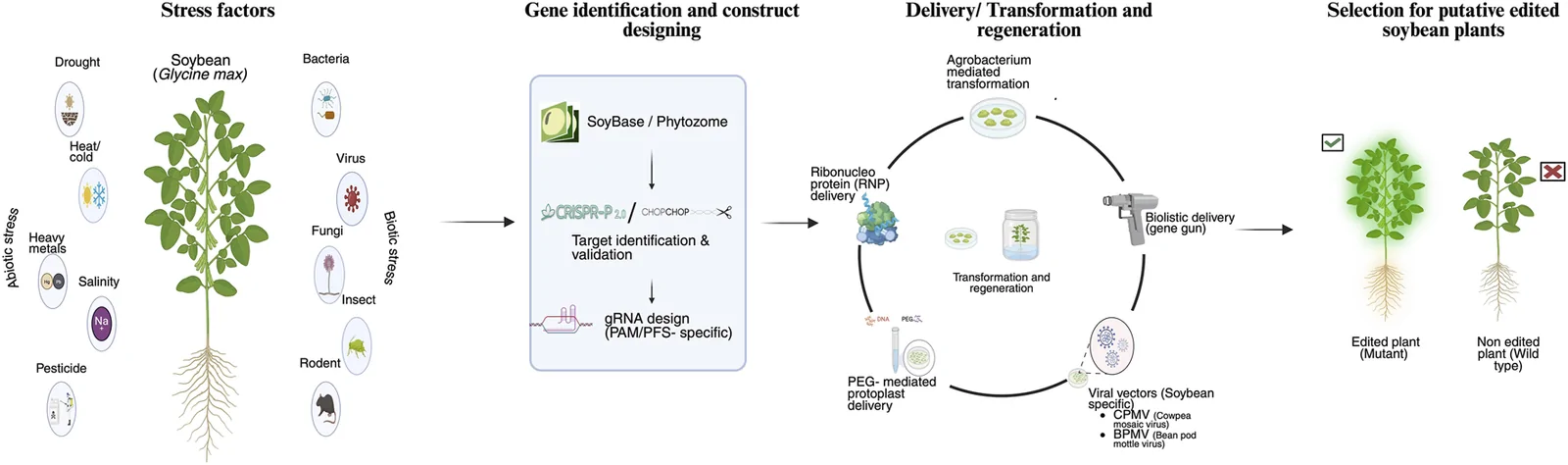
Schematic pipeline for CRISPR-based genome editing in soybean (*Glycine max*). The pipeline is organized into four stages: (i) Gene identification and construct designing, involving gene/trait selection, *in silico* target analysis using soybean genome v2.1, PAM/PFS-specific gRNA design, and recommended bioinformatic tools including CRISPR-P, CHOPCHOP, Benchling, CRISPOR, gRNAde, SoyBase, and GMO Genome Browser; (ii) gRNA design, illustrating distinct guide RNA architectures and PAM/PFS requirements for each CRISPR-Cas system alongside the off-target prediction tools Cas-OFFinder and CCTop; (iii) Soybean delivery methods, encompassing *Agrobacterium*-mediated transformation, biolistic gene gun, PEG-mediated protoplast delivery, ribonucleoprotein (RNP) delivery, and soybean-specific viral vectors (CPMV and BPMV); and (iv) Outcome analysis, comprising molecular analysis, edit profiling, off-target analysis, expression analysis, and phenotypic evaluation. Created in BioRender. Rajendran (2026) https://biorender.com/b2i15b2.

A critical challenge in designing CRISPR constructs is the potential occurrence of off-target cleavage events, wherein Cas9 introduces double-strand breaks at unintended genomic sites, leading to detrimental phenotypic consequences (Guo et al., 2023b). Cas9 can tolerate up to three mismatches between sgRNA and genomic DNA, leading to off-target effects due to sgRNA sequences (Fu et al., 2014; Hsu et al., 2013). *In silico-based* detection tools such as Cas-OFFinder, CCTop (Consensus Constrained TOPology prediction), CROP-IT (CRISPR/Cas9 Off-target Prediction and Identification Tool), DeepCRISPR, and Elevation software remain the foremost choice for identifying potential off-target sites. The tools offer alignment-based and scoring-based models that are freely accessible to the research community (Guo et al., 2023b). Trinh et al. (2022) employed CCTop to validate sgRNAs for a soybean-based genome editing system and successfully finalized three high-specificity sgRNAs for downstream editing applications. However, computational methods do not fully account for the complicated intranuclear microenvironment and require complementary experimental validation. Currently, using cell-free sequencing approaches such as Digenome-Seq provides deep genomic coverage capable of detecting InDel events at frequencies as low as 0.1% (Kim et al., 2015).

Due to the recalcitrant nature of soybean explants, the transformation protocol represents yet another critical bottleneck in soybean genome editing. *Agrobacterium tumefaciens-*mediated and biolistic transformation are two principal platforms for stably integrating the constructs into the soybean genome (Xu et al., 2022). Although overall efficiency is considered relatively low, several co-cultivation parameters can be tweaked in order to enhance the transformation frequency. The most critical parameters are maintenance of proper co-cultivation conditions, *Agrobacterium* infection conditions, adequate antioxidant supplementation, and genotype-specific plant growth regulator combinations (Yamada et al., 2012; Li S. et al., 2017). Karthik et al. (2020) demonstrated the supplementation of a nitric oxide donor such as sodium nitroprusside to enhance the genetic transformation of soybean. Recently, considerable progress has been made in improving transformation efficiency and reducing genotype dependency through the use of plant morphogenic regulators such as *Baby boom* (BBM) and *WUSCHEL2* (*WUS2*) genes and *GROWTH-REGULATING FACTORS* (GRF) genes (Gordon-Kamm et al., 2019; Debernardi et al., 2020; Kong et al., 2020). Notably, Alok et al. (2025) reported that co-expression of *WUS2* and isopentenyltransferase *(IPT)* genes achieved transformation efficiencies of 22.3% in Williams 82 and Bert soybean varieties without requiring exogenous hormones or selection agents. It successfully generated heritable transgenic events within 9 weeks to 11 weeks with 20% mutation efficiency. These collective developments reflect the steady progress being made toward overcoming the longstanding transformation barriers in soybean and exploring new avenues to enhance the gene construct uptake, thereby accelerating the generation of genome-edited soybean varieties (Yao et al., 2023).

## 4 Application of CRISPR/Cas for soybean

Soybean is one of the most valuable oilseed crops, yet like other crops, it faces challenges from both biotic and abiotic stresses. It also has limitations in nutritional and agronomic traits. With the advent of CRISPR/Cas systems, these traits can be precisely manipulated to create improved varieties. The following section highlights how CRISPR/Cas9 is being applied to edit soybean genes for enhanced performance across multiple attributes.

### 4.1 Advancement of soybean resistance mechanisms to biotic stress factors

Biotic stress is a major constraint on crop productivity and poses a serious challenge to global food security. Biotic factors include pathogens such as bacteria, viruses, fungi, nematodes, etc. Among these are 29 fungal, 6 bacterial, 18 viral, 6 nematodal, and 3 mycoplasmal diseases reported in soybean (Akem, 1996), and their estimated yield losses are mentioned in Table 3. A number of control measures are being developed and adopted to manage different diseases, as chemical control of diseases can be costly and negatively impact the environment (Roth et al., 2021). For instance, MYMIV accounts for 80% yield loss in soybean (Yadav et al., 2015), and no successful gene editing strategy has been developed for soybean. It is therefore imperative to identify key players for susceptibility genes and secondary metabolite pathways that could give rise to resistant cultivars, limiting the yield loss by this virus, especially in India. Consequently, genetic development against plant disease is crucial to achieving sustainable agricultural production. In this regard, genome editing has emerged as a transformative biotechnological approach that facilitates precise, unambiguous, and targeted genome modifications, thereby enabling the enhancement of disease resistance and accelerating resistance breeding programs.

**TABLE 3:** List of different biotic stresses and their impact on soybean yield (%).

Biotic stress	Pathogen/pest	Estimated yield loss (in %)	Reference
Tobacco cutworm (*Spodoptera litura)*	Insect	0.67 to 17.71	Sundar et al. (2020)
Whitefly (*Bemisia tabaci*)	Insect	18.12–27.28%	Harish et al. (2024)
Yellow mosaic disease	Virus	Up to 85.7%	Amrate (2024)
Anthracnose/pod blight (*Colletotrichum/Phomopsis spp*.)	Fungus	64.8–100%	Amrate et al. (2023)
Rust (*Phakopsora pachyrhizi*)	Fungus	30–100%	Sharma et al. (2014)
Collar rot (*Athelia rolfsii*)	Fungus	30–40%	Sharma et al. (2014)
Charcoal rot (*Macrophomina phaseolina*)	Fungus	55.6–100% (severe cases)	Amrate et al. (2020)
Rhizoctonia root rot (*Rhizoctonia spp.*)	Fungus	30–35%	Sharma et al. (2014)
Fusarium seedling (*Fusarium spp.*)	Fungus	60–64%	Sharma et al. (2014)
Soybean cyst nematode (*Heterodera glycines*)	Nematode	30–100%	Sharma et al. (2014)

#### 4.1.1 Virus resistance

Soybean mosaic virus (SMV) is one of the most economically damaging viral pathogens affecting global soybean production. It causes significant reductions in seed quality and yield. The viral *HC-Pro* gene functions as a key pathogenicity factor by regulating SMV viral translation, replication, intracellular trafficking, cell-to-cell movement, and inhibition of post-transcriptional gene silencing (PTGS). This consequently induces silencing of the chalcone synthase gene and causes characteristic seed-coat mottling in infected soybean plants.

Gao et al. (2024) used multiple CRISPR/CasRx antiviral systems, namely CRISPR/CasRx(+), CRISPR/CasRx(−), and CRISPR/CasRx(+/−). Amongst these, using a CRISPR/CasRx(+) strategy to target the positive-sense strand of the SMV *HC-Pro* gene exhibited the highest level of SMV resistance, with the highest proportions of resistant plants recorded in both progenies (43.96%) and T2 generations (55.56%). This suggests that direct silencing of important viral RNA components can provide highly efficient and rapid resistance against SMV compared to other conventional breeding approaches. The *Rsc4* locus is the only dominant genetic locus conferring SMV resistance in soybean and harbors three nucleotide-binding domain leucine-rich repeat-containing (NLR) candidate genes, including *Rsc4-1, Rsc4-2*, and *Rsc4-3.* Of these, *Rsc4-3* alone confers functional resistance to SMV. Yin et al. (2021) used CRISPR/Cas9-mediated knockout to validate the function of *Rsc4-3*, and the resultant mutant lines exhibited characteristic mosaic and leaf wrinkling symptoms following SMV challenge inoculation. This confirmed that *RSC4-3* is essential for resistance, and its loss of function impairs the ability of the plant to recognize and respond to viral infection. By comparing both *HC-Pro*-based targeting (S-gene-mediated) and *Rsc4-3*-mediated defense (R-gene-mediated), the S-gene-mediated defense response is more reliable and effective than others, due to the high risk of resistance breakdown and viral evolution in R-gene-mediated defense.

In addition to direct viral and resistance gene targeting, metabolic engineering of the isoflavonoid biosynthesis pathway has been explored as an alternative strategy for enhancing SMV resistance. Isoflavonoids are important secondary metabolites with documented antiviral properties in soybeans, and isoflavone synthase (IFS) is a key enzyme in isoflavonoid synthesis that shares a common substrate pool with flavanone-3-hydroxylase *(F3H)* and flavone synthase II *(FNS II)*. IFS catalyzes the production of daidzein and naringenin and related defense compounds (Subramanian et al., 2006). Zhang et al. (2020) applied a CRISPR/Cas9 multiplex approach to simultaneously silence competing branch pathway genes such as *GmF3H1*, *GmF3H2*, and *GmFNSII-1,* thereby redirecting metabolic flux toward isoflavone accumulation. Metabolic analysis of the edited plants revealed a significant increase in total isoflavone content relative to the wild-type control, which was accompanied by an approximately 33% reduction in SMV coat protein content. This further indicated that elevated isoflavone levels enhanced the plant capacity to resist viral infection.

#### 4.1.2 Insect resistance

Flavonoids constitute a critical class of secondary metabolites in soybean. They function as defense compounds against chewing types of insects that inflict substantial yield losses and compromise seed quality. The marker gene for QTL-M is *Glyma.07g110300* (LOC100775351), which encodes UDP-glycosyltransferase (*UGT*). Overexpression of UDP-glycosyltransferase (UGT) alters flavonoid profiles and reduces the accumulation of bioactive defense metabolites, thereby sensitizing plants to insect herbivory and suggesting its role as a negative regulator of flavonoid-mediated defense. Consequently, CRISPR/Cas9-mediated loss-of-function mutations in *UGT* have been shown to markedly increase the levels of daidzein and formononetin, providing enhanced resistance to *Helicoverpa armigera* and *Spodoptera litura.* The real-time transcriptional profiling of mutant lines revealed upregulation of key flavonoid biosynthetic genes, including chalcone synthase (*GmCHS*), chalcone reductase (*GmCHR*), and cytochrome P450 monooxygenase (*GmCYP81E11*). The expression of defense-associated genes was also enhanced, which validated the mechanistic basis of improved insect resistance (Zhang et al.*,* 2022). In addition, calcium-dependent protein kinase *GmCDPK38* has been functionally characterized as a dual regulator of flowering time and insect resistance in soybean. In another study, targeted mutagenesis of *GmCDPK38* resulted in delayed flowering and increased resistance to common cutworms under both short and long-day photoperiods (Li et al., 2022). The rationale for targeting *UGT* and *GmCDPK38* lies in the strategic enhancement of endogenous defense pathways, thereby conferring broad-spectrum resistance, rather than directly targeting insects or pests.

#### 4.1.3 Oomycetes

*Phytophthora sojae* is a soil-borne oomycete pathogen responsible for root and stem rot in soybean, which leads to severe yield losses. The resistance of soybean to this pathogen is primarily mediated by *Rps* genes, which recognize pathogen avirulence effectors encoded by *Avr1b* in a gene-for-gene manner and trigger hypersensitive responses that result in failure of infection (Song et al., 2013). Among these, the *Avr1b* effector of *P. sojae* encodes an RXLR protein recognized by soybean cultivars carrying *Rps1b* or *Rps1k,* thereby conferring immunity. Gu et al. (2021) employed a *Phytophthora*-specific CRISPR/Cas9 system to generate knockout and knock-in mutants of the *Avr1b-1* gene to validate its functional role in soybean for understanding the molecular basis of *Rps/Avr* interaction. The knockout mutants overcame *Rps1b-*mediated resistance, which led to susceptibility, and reintroduction of *Avr1b* ^*sgR*^ into the knockout mutant restored avirulence in mutants. These results confirmed the essential role of the *Avr1b-1* gene in defense activation. Similarly, Arsenault-Labrecque et al. (2022) validated the interaction between *Avr* effectors and resistance gene *Rps8*, which targeted two candidate genes, *Avh37* ^*45C*^ and *Avr3a* ^*45*C^ of *P. sojae,* resulting in susceptibility in mutant plants. CRISPR/Cas9-mediated mutation of *Avr3a* ^*45*C^ introduced a single nucleotide deletion within the Asp-Glu-Glu-Arg motif, producing a truncated protein that abolished recognition and resulted in virulence. Ochola et al. (2020) further demonstrated the importance of temporal regulation of avirulence (*Avr*) effectors by replacing the native promoter of *PsAvr3b* with *PsACT* (actin constitutive expression), *PsXEG1* (early expression), and *PsNLP1* (later expression) using the CRISPR/Cas9 technique. The altered expression patterns significantly reduced *Avr* activity, leading to susceptibility in *Rps3b*-carrying soybean plants, whereas constitutive expression remained avirulent.

*P. sojae* secretes some effectors that actively promote susceptibility in plants apart from those involved in *Avr-Rps* interactions. For instance*, PsAvh52* recruits the *GmTAP*1 gene in the nucleus, deacetylating histones H2A and H3 to activate susceptibility genes (Li et al., 2018). Liu T. et al. (2023) constructed a *pFGC5941-Cas9-GmTAP1* vector targeting exon 4 of *GmTAP1*, which provides resistance against three different *P. sojae* strains, including P231, P233, and P234, with no significant effect on agronomic characteristics. Ma et al. (2020) disrupted the *Gmlmm2-1* gene, which regulates tetrapyrrole and chlorophyll biosynthesis. Knockout mutants accumulated reactive oxygen species (ROS), triggered hypersensitive responses, and showed enhanced resistance, although they showed reduced chlorophyll content and necrotic leaf lesions due to pathway disruption. Luo et al. (2024) identified the stress-responsive gene *GmARM,* which negatively regulates defense signaling and contributes to abiotic stress. CRISPR/Cas9-mediated disruption of *GmARM* enhanced resistance of soybean to *P. sojae* and simultaneously conferred tolerance to alkali and salt stress, highlighting its dual role as a negative regulator of multi-stress responses.

#### 4.1.4 Nematodes

The soybean cyst nematode SCN (*Heterodera glycines*) represents the most economically devastating pathogen of soybean. The resistance is largely conferred by the *Rhg1* locus, which encodes the α-SNAP protein. The functional studies have revealed that α-SNAP interacts with syntaxin proteins to mediate SCN resistance (Dong et al., 2020). They employed CRISPR/Cas9 to generate targeted deletions in four syntaxin homologs (*Syn12, Syn13, Syn16*, and *Syn02*). While single knockouts of the syntaxin gene showed less impact on SCN resistance, double knockouts increased susceptibility, and quadruple knockouts abolished resistance, thereby confirming the essential role of syntaxin in defense. Further validation was achieved using a dual-guide CRISPR/Cas9 system for targeted knockout of *GmSNAP02* in transgenic hairy roots of Peking composite plants. These two dual gRNAs were designed to target the 5′ and 3′ regions of the *GmSNAP02* gene, where the first gRNA CRISPR/Cas9-GmSNAP02-T4 + T3 targeted exon 1 and exon 5, and the second gRNA CRISPR/Cas9-GmSNAP02-T5 + T7 targeted two sites in exon 9 and the 3′-UTR region. After challenge inoculation was performed, mutant plants showed a significant reduction in cyst numbers compared to plants transformed with empty vector controls after 28 days post-inoculation. This demonstrated the functional contribution of *GmSNAP02* to SCN resistance (Usovsky et al., 2023). Similarly, Butler et al. (2021) knocked out c-γSNAP (*Glyma.15G191200*), resulting in mutant plants that showed increased susceptibility to nematodes.

#### 4.1.5 Fungus

The 14-3-3 protein encoded by the *Glyma 05g29080* gene is a regulatory molecule involved in multiple plant processes, including nodulation, defense, signaling pathways, production of reactive oxygen species, cell wall, and guard cell regulation. Zhang et al. (2023) validated its functionality by CRISPR/Cas9-mediated knockout.

Subsequent infection of *S. sclerotiorum* on the edited plant revealed a significant increase in fungal infection severity alongside a marked reduction in nodulation compared to wild-type controls. These collectively confirmed the role of *Glyma 05g29080 in* defense responses and symbiotic nodule development in soybean. Powdery mildew represents another significant fungal threat to soybean production, which is caused by *Erysiphe diffusa.* The mildew resistance locus O (*MLO*) gene family encodes negative regulators of plant defense that promote susceptibility to powdery mildew infection by suppressing papilla formation, reducing ROS accumulation, and modulating cell death responses. In Vietnamese soybean cultivar DT26, the *GmMLO* gene was targeted by using dual-sgRNA CRISPR/Cas9 constructs, and through multiplex editing, it generated triple (*Gmmlo02/Gmmlo19/Gmmlo23*) and quadruple (*Gmmlo02/Gmmlo19/Gmmlo20/Gmmlo23*) knockout mutant lines. When the mutant lines were challenged with *Erysiphe diffusa,* they demonstrated that all mutant lines exhibited measurable resistance to the pathogen, with a reduction in the severity of powdery mildew by 36.4% compared to wild-type plants (Bui et al., 2023).

Overall, these studies highlight the versatility of CRISPR-based genome editing in dissecting gene function and engineering resistance against a broad range of biotic stresses in soybean. By targeting viral genomes, host susceptibility genes, secondary metabolite biosynthesis pathways, and disease resistance gene networks, CRISPR-based approaches provide a precise and efficient platform for developing resilient soybean germplasm with durable resistance to diverse pathogens. Some of the important applications of CRISPR systems in biotic stress are listed in Table 4.

**TABLE 4:** Applications of CRISPR/Cas9 for biotic stress management.

Target gene	Pathogen/insect	Gene function	CRISPR editing platform	Delivery method	CRISPR reagent	SDN type	Transgene-free	Research outcome	Reference
Insects									
*GmCDPK38*	*Argotis epsilon*	Photoperiod regulation and defense against common cutworm (CCW)	CRISPR/Cas9	*Agrobacterium*-mediated	sgRNA + Cas9	SDN-1	No	Flowering and enhanced resistance to CCW	Li et al. (2022)
*GmUGT*	*Helicoverpa armigera* and *Spodoptera litura*	Encodes a UDP-glycosyltransferase (UGT), a susceptibility factor	CRISPR/Cas9	*Agrobacterium*-mediated	sgRNA + Cas9	SDN-1	Yes	Increased resistance against leaf chewing insects in soybean	Zhang Y. et al. (2022)
Oomycetes									
*GmTAP1*	*Phytophthora sojae*	Acetylates histones H2A and H3, which increases the transcription of susceptibility genes	CRISPR/Cas9	*Agrobacterium*-mediated	sgRNA + Cas9	SDN-1	Yes	Enhanced resistance against different strains of *P. sojae*	Liu L. et al. (2023)
*PsAvr3b*	*Phytophthora sojae*	PsAvr3b encodes an Avr effector that is recognized by the resistant gene Rps3b and activates an immune response	CRISPR/Cas9	Transformation (oomycete system)	sgRNA + Cas9	SDN-1	No	Functional validation of PsAvr3b gene	Ochola et al. (2020)
*Avr1b-1*	*Phytophthora sojae*	Avr1b encodes an RXLR effector protein required for infection	CRISPR/Cas9	Particle bombardment/transformation	sgRNA + Cas9	SDN-2	No	Interaction between Avr1b-1 and Rsp gene results in resistance in plants	Gu et al. (2021)
*GmLMM2*	*Phytophthora sojae*	Biosynthesis of tetrapyrrole and light-dependent defense in soybean	CRISPR/Cas9 (inference)	*Agrobacteriu*m-mediated	sgRNA + Cas9 + HDR donor	SDN-1	No	Confirm GmLMM2 gene provides resistance against *Phytophthora sojae*	Ma et al. (2020)
*SGF14c*	*Sclerotinia sclerotiorum*	Defense against *Sclerotinia sclerotiorum and in* nodulation	CRISPR/Cas9	*Agrobacterium*-mediated	sgRNA + Cas9	SDN-1	No	CRISPR-Cas9 mutation showed reduced resistance to S. *sclerotiorum* and reduced nodulation	Zhang et al. (2023)

Target gene	Pathogen	Gene function	CRISPR editing platform	Delivery method	CRISPR reagent	SDN type	Transgene-free	Research outcome	Reference
Fungus									
*GmMLO*	*Erysiphe diffusa*	Membrane-associated protein that negatively regulates plant defense	*CRISPR/Cas9 (dual-sgRNA, multiplex editing)*	*Agrobacterium-mediated*	sgRNA + Cas9 multiplexing	SDN-1	No	Increasing resistance against powdery mildew	Bui et al. (2023)
*GmARM*	*Phytophthora* root rot	Act as a negative regulator of stress responses	CRISPR/Cas9	A*grobacterium-*mediated transformation	CRISPR reagent	SDN-1	No	Resistance to saline-alkali stress and *Phytophthora* root rot infection	Luo et al. (2024)
Nematode									
γ-SNAP	*Heterodera glycines*	Encodes the γ-SNAP protein, which is involved in vesicle trafficking	CRISPR/Cas9	*Agrobacterium rhizogenes* (hairy root)	sgRNA + Cas9	SDN-1	No	Gene editing revealed a role for Glyma.15G191200 of cqSCN-006 in SCN resistance	Butler et al. (2021)
*GmSNAP02*	*Heterodera glycines*	Vesicle trafficking and membrane fusion processes	CRISPR/Cas9	*Agrobacterium rhizogenes* (hairy root transformation)	sgRNA + Cas9	SDN-1	No	Significant reduction in cyst number	Usovsky et al. (2023)
Virus									
*HC-Pro gene*	Soybean mosaic virus (SMV)	Acting as the inhibitory apparatus for post-transcriptional gene silencing (PTGS) by blocking the production of cellular siRNAs and microRNAs	CRISPR/CasRx	*Agrobacterium*-mediated	sgRNA + Rx plasmid	SDN-1	No	Resistance to soybean mosaic virus	Gao et al. (2024)
*GmF3H, GmF3H2*, and *GmFNSII-1*	Soybean mosaic virus	Key enzyme in isoflavonoid synthesis	CRISPR/Cas9	*Agrobacterium*-mediated	sgRNA + Cas9	SDN-1	No	Increase isoflavone content and resistance to SMV	Zhang et al. (2020)

Gene abbreviations: γ-SNAP, soluble N-ethylmaleimide-sensitive factor attachment protein; GmARM, *Glycine max* armadillo repeat protein; GmF3H: *Glycine max* flavanone 3-hydroxylase; FNS, flavone synthase; HC-Pro, helper component-proteinase; GmMLO, *Glycine max* mildew locus O; GmLMM2, *Glycine max* lesion mimic mutant 2; SGF14c, soybean G-box factor 14-3-3c; Avr, avirulence (gene); GmTAP, *Glycine max* transporter associated with antigen processing; GmUGT, UDP-glycosyltransferase; GmCDPK38, *Glycine max* calcium-dependent protein kinase 38.

### 4.2 Improvement of soybean resilience to abiotic stress conditions

Abiotic stress has become an increasingly serious threat to crop production worldwide, driven largely by climate change and industrialization. Drought, salinity, and extreme temperatures are among the most prevalent abiotic stresses, and soybean is particularly vulnerable to all of them. These conditions can disrupt soybean growth at any stage, ultimately causing yield losses of more than 50% of its production potential (Fang et al., 2026). The yields attributable to major abiotic stresses in soybean are summarized in Table 5.

**TABLE 5:** List of different abiotic stresses and their impact on soybean yield (%).

Abiotic stress	Estimated yield loss (in %)	Reference
Drought	25–50	Zou et al. (2012)
Cold	24	Staniak et al. (2021)
Heavy metals	19–20	Shaffique et al. (2023)
Heat	17	Zhao et al. (2017)

#### 4.2.1 Salt tolerance

Soil salinization affects approximately 20% of the world’s cultivated land and 33% of irrigated agricultural land, which significantly limits soybean productivity. CRISPR/Cas9-based functional studies have identified several key genes involved in ionic homeostasis, ABA signaling, and antioxidant defense under salt stress. Knockout mutants of *GmAITR* (ABA-induced transcription repressor), which were generated using CRISPR/Cas9, exhibited enhanced salt tolerance, with higher survival rates and improved growth under saline conditions without compromising growth in non-saline soil. These edited lines also displayed lower MDA and H_2_O_2_ levels under salt stress, indicating reduced oxidative damage (Wang et al., 2021). Chavhan et al. (2025) confirmed that *GmAITR* mutants displayed improved K^+^/Na^+^ ratios and enhanced salinity resilience, which established *GmAITRs* as negative regulators of salt stress response. Notably, *gmaitr23456* quintuple mutants showed comparably higher salt tolerance and ABA responses than *gmaitr36* double mutants. This could likely be attributed to functional redundancy among *GmAITR* paralogs, suggesting that targeting a subset of *GmAITR* members may be sufficient for achieving meaningful tolerance improvements.

Ion transporter genes have also been functionally validated under salt stress conditions. CRISPR/Cas9-mediated knockout of *GmSOS1* that codes for a plasma membrane Na^+^/H^+^ antiporter resulted in elevated Na^+^ accumulation and disrupted ionic homeostasis. This confirmed the essential role of this transporter in Na^+^ exclusion via the conserved SOS pathway (Yazar et al., 2025). Similarly, knocking out the potassium channel gene *GmAKT1* reduced K^+^ content and increased salt sensitivity, while overexpression lines showed enhanced K^+^ uptake and tolerance mediated by the upstream *GmCBL9–GmCIPK6* regulatory complex (Feng et al., 2025). Together, these findings indicate that while *GmSOS1* primarily governs Na^+^ exclusion, *GmAKT1* operates through K^+^ retention, and both mechanisms are independently essential for maintaining ion homeostasis under salinity stress. Zhou et al. (2025) highlighted the synergistic roles of *GmNHX1*, *GmSOS1*, *GmHKT1*, and *GmCLC1* in maintaining Na^+^/K^+^/Cl^−^ balance by integrating CRISPR, Omics, and microbial strategies for developing salt-tolerant soybean varieties.

NAC transcription factors have also been implicated in soybean salt stress responses. Overexpression of *GmNAC03* enhanced germination and seedling tolerance under saline conditions, accompanied by elevated SOD, POD, and CAT activities, reduced MDA levels, and amino acid metabolic reprogramming (Shi et al., 2025a). In *Arabidopsis, GmNAC035* overexpression activated SOS, calcium, and ABA signaling pathways, helping to mitigate oxidative damage under salt stress (Shi et al., 2025b), while *GmNAC018* overexpression in transgenic soybean improved chlorophyll content, biomass, and Na^+^ exclusion (Ma et al., 2026).

Although these overexpression studies have provided valuable insights into NAC transcription factor function under salt stress, CRISPR-based knockout validation remains limited for most genes, and further studies could help clarify their precise endogenous roles in soybean salinity responses. The role of *GmNHL1* was validated through both overexpression and CRISPR knockout approaches, in which overexpression lines exhibited increased chlorophyll and proline levels, elevated peroxidase activity, and reduced MDA and superoxide anion content. Field-level comparisons further confirmed superior growth of edited lines over wild-type plants under salt stress, which supported *GmNHL1* as a strong candidate for salt-tolerant soybean breeding (Liu L et al., 2023). While CRISPR/Cas9-based studies have successfully validated several candidate genes governing ionic homeostasis and oxidative stress responses in soybean, a critical gap remains in field-level evaluation and multi-gene stacking strategies. These limitations could be effectively addressed for translating laboratory findings into salt-tolerant soybean varieties suitable for real-world saline environments.

#### 4.2.2 Drought tolerance

Drought stress is one of the primary abiotic constraints limiting soybean growth and productivity. *GmHdz4* is a plant-specific HD-Zip I transcription factor that was knocked out by using CRISPR/Cas9 in the Tianlong No. 1 cultivar. The mutants exhibited significantly better drought tolerance with improved root architecture, increased total root length, root surface area, and root tip number, leading to enhanced water uptake efficiency under drought stress. The edited plants also exhibited increased osmolyte accumulation and elevated antioxidant activities, thereby reducing ROS-induced cellular damage. In contrast, overexpression of *GmHdz4* reduced drought tolerance, identifying it as a negative regulator whose suppression improves soybean resilience under water deficit (Zhong et al., 2022). Unlike *GmHdz4*, overexpression of *GmPM35* in both *Arabidopsis* and soybean enhances drought tolerance, indicating that positive and negative regulatory mechanisms operate in parallel to fine-tune drought responses in soybean. The transgenic *GmPM35* plants showed improved photosynthetic efficiency, root development, relative water content, chlorophyll content, and antioxidant capacity under water deficit. *GmPM35* overexpression lines exhibited reduced ROS accumulation and lipid peroxidation, alongside stronger induction of drought-responsive genes including *DREB2A*, *RD17*, *RD26*, and *CBF3.* This suggests that *GmPM35* functions as a positive regulator of drought-response signaling that complements the antioxidant defense mechanisms identified in *gmhdz4* mutants (Wang et al., 2025b). Extending these findings to an OVATE family protein, a similar observation was found by Zhang et al. (2025). They demonstrated that *GmOFP8* similarly functions as a positive regulator of drought tolerance in soybean. CRISPR/Cas9-mediated knockout of *GmOFP8* resulted in severe wilting, reduced relative water content, and a 20% survival rate under prolonged drought compared to an approximately 70% survival rate in overexpression lines and a 33% survival rate in wild-type plants. Knockout mutants accumulated higher H_2_O_2_ and MDA levels with reduced POD activity, while overexpression lines showed upregulation of drought-responsive markers *GmRD26*, *GmDREB1*, and *GmMYB14*, along with enhanced antioxidant gene expression. Compared to *GmPM35, GmOFP8* additionally integrates nodulation and brassinosteroid signaling through its physical interaction with *GmSK2*. It is a glycogen synthase kinase 3-like kinase that negatively regulates both drought tolerance and nodulation, thereby suggesting a broader regulatory role beyond stress adaptation (Zhang et al., 2025).

Zhang et al. (2026) identified *GmMRFL*, a MORN-motif repeat protein that concurrently promotes photoperiod-dependent flowering and enhances drought resilience through stomatal regulation, ABA signaling, and ROS suppression. The transcription factor GmERF011 specifically activates the Hap1 promoter variant of *GmMRFL*, while *GmMRFL* interacts with *GmANK2* to establish the GmERF011–GmMRFL/GmANK2 regulatory module. The ABA-mediated stomatal regulation observed for *GmMRFL* aligns with the antioxidant and osmotic adjustment mechanisms identified in *GmHdz4*, *GmPM35*, and *GmOFP8* studies, collectively pointing to a convergent drought-response network in soybean that operates across transcriptional, hormonal, and signaling levels. Phenotypic analyses of hairy roots validated the role of *GmERF011* in enhancing drought tolerance, establishing *GmMRFL* as a promising target for multi-trait engineering in soybean precision breeding (Zhang et al., 2026). While CRISPR/Cas9-based studies have provided robust functional evidence for negative regulators such as *GmHdz4* and positive regulators such as *GmOFP8* in soybean drought tolerance, several studies, including those on *GmPM35* and *GmMRFL*, still rely predominantly on overexpression for functional validation. Field-level evaluation of edited lines under variable drought regimes remains limited, and future breeding strategies should prioritize multiplex editing of complementary drought tolerance pathways to develop broadly resilient soybean cultivars.

#### 4.2.3 Heat tolerance

Heat stress is an increasingly significant abiotic constraint on soybean productivity, particularly during reproductive stages in which high temperatures adversely affect pollen viability, seed set, and overall yield. CRISPR/Cas9-based and complementary functional studies have identified key molecular regulators of thermotolerance in soybean, operating through distinct but interconnected mechanisms. Huang et al. (2019) demonstrated that *GmHsp90A2*, encoding heat shock protein 90 (HSP90), functions as a positive regulator of thermotolerance in soybean. *GmHsp90A2* overexpression lines enhanced heat tolerance by reducing chlorophyll degradation, electrolyte leakage, and lipid peroxidation under 38 °C heat stress. These lines also upregulated *HsfA1s*, *Hsp70s*, and antioxidant genes including *GmGST* and *GmCAT*. *GmHSP70* was characterized in the same study as a gene that functions cooperatively within the same heat-responsive pathway, suggesting that HSP70 and HSP90 family members act in a coordinated manner to maintain protein homeostasis under heat stress.

Hou et al. (2024) identified a distinct but complementary mechanism through *GmBSK1*, which is a brassinosteroid-signaling kinase. CRISPR/Cas9-mediated knockout of *GmBSK1* increased ROS accumulation and heat sensitivity, while overexpression lines enhanced ROS scavenging, improved physiological traits, and maintained yield under heat stress. *GmBSK1* antagonizes the *GmGSK1–GmBES1.5* interaction by promoting *GmGSK1* plasma membrane localization and releasing *GmBES1.5* transcriptional activity. *GmBES1.5* subsequently binds E-box motifs in the promoters of heat-responsive genes including *GmPP2C27* and *GmPLDα1*, thus upregulating their expression through the *GmBSK1–GmGSK1–GmBES1.5* regulatory module. These findings indicate that soybean thermotolerance is governed by at least two parallel regulatory axes, including chaperone-mediated protein homeostasis and brassinosteroid-dependent transcriptional reprogramming. Both of them show ROS detoxification and antioxidant defense as common downstream outcomes. Field-level evaluation of heat-tolerant edited lines remains limited, and future breeding strategies should explore multiplex editing targeting both chaperone and hormonal regulatory pathways to develop broadly heat-resilient soybean cultivars.

#### 4.2.4 Heavy metal stress

Heavy metal contamination poses a serious threat to soybean productivity and food safety. While aluminum (Al) toxicity remains a major constraint on acidic soils, cadmium (Cd) accumulation disrupts key physiological processes including photosynthesis, antioxidant defense, and nutrient uptake. Transcription factors such as bZIP, DREB, and NAC show potential for improving metal tolerance, but their roles in soybean remain underexplored. CRISPR/Cas9 offers promising solutions for precise functional validation and improvement of metal tolerance genes in soybean breeding. Ma J. et al. (2021) conducted comparative root transcriptome analysis between Cd-sensitive (KUAI) and Cd-tolerant (KAIYU) soybean cultivars. They identified antioxidant defense, heavy metal sequestration, and phenylpropanoid biosynthesis as primary tolerance mechanisms, in which POD, CAT, and SOD activities were significantly higher in KAIYU under Cd stress. The functional validation was carried out through overexpressing *GmHMA5*, a heavy metal-transporting ATPase, in hairy roots of Cd-sensitive KUAI. This significantly improved root Cd tolerance, confirming the role of P1B-ATPase-mediated sequestration in detoxification. Gong et al. (2026) identified *GmMYB84*, an R2R3-MYB transcription factor, as a key regulator of Cd tolerance through CRISPR/Cas9-mediated functional analysis. Overexpressing *GmMYB84* in *Arabidopsis* enhanced Cd tolerance through improved germination, root length, chlorophyll content, and flavonoid levels, alongside reduced oxidative damage markers. Conversely, CRISPR/Cas9-generated *myb84* knockout soybean lines exhibited severe growth inhibition and elevated oxidative stress under Cd exposure. Mechanistically, *GmMYB84* binds the MBSI cis-element in promoters of *Glyma.01G172700* and *Glyma.02G042500*, linking Cd tolerance to phenylpropanoid-mediated secondary metabolism and cell wall reinforcement.

Cai et al. (2024) revealed a distinct tolerance mechanism through rhizosphere microbiome modulation. CRISPR/Cas9 double knockout of *GmAMT2.1/2.2* ammonium transporter genes resulted in pronounced Cd sensitivity and reduced NH_4_^+^-N uptake, while overexpression lines showed enhanced tolerance and lower Cd accumulation. *GmAMT2.1/2.2* genes alter root exudate composition to recruit beneficial microorganisms including *Tumebacillus*, *Alicyclobacillus*, and *Penicillium*. The synthetic microbial communities constructed from these organisms significantly reduced Cd content and upregulated genes involved in phenylpropanoid biosynthesis and heavy metal detoxification. When compared to *GmMYB84*-mediated tolerance operating through transcriptional activation of secondary metabolism, *GmAMT2.1/2.2*-dependent tolerance introduces an additional regulatory layer through microbiome assembly. While CRISPR/Cas9 has provided robust functional evidence for *GmMYB84* and *GmAMT2.1/2.2*, validation of sequestration-related genes such as *GmHMA5* remains limited to overexpression approaches. Field-level evaluation under variable Cd concentrations remains limited, and future strategies should target multiplex editing of complementary tolerance mechanisms to develop broadly metal-resilient soybean cultivars. Table 6 summarizes key CRISPR/Cas9-based functional studies targeting abiotic stress tolerance in soybean, highlighting gene targets, editing approaches, and research outcomes across salt, drought, heat, and heavy metal stress conditions.

**TABLE 6:** Applications of CRISPR/Cas9 for abiotic stress management in soybean.

Target gene	Gene function	CRISPR system used	Delivery method	CRISPR reagent	SDN type	Transgene-free	Research outcome	Reference
Salt stress								
*GmAITR*	ABA-induced transcription repressor; negative regulator of ABA signaling and salt stress response	CRISPR/Cas9	*Agrobacterium*-mediated	sgRNA + Cas9	SDN-1	No	Knockout mutants showed enhanced salt tolerance, higher survival rates, improved K+/Na+ ratio, reduced MDA and H_2_O_2_ levels	Wang et al. (2021), Chavhan et al. (2025)
*GmARM*	Armadillo repeat protein; stress-regulatory gene involved in ABA signaling	CRISPR/Cas9	*Agrobacterium*-mediated	sgRNA + Cas9	SDN-1	No	Knockout mutants showed higher survival under salt (30–43% vs. 3%) and alkali stress (43–74% vs. 3%), and medium-to-high resistance against *Phytophthora sojae*	Luo et al. (2024)
*GmNHL1*	Non-host resistance 1/Harpin-induced 1-like; positive regulator of salt tolerance	CRISPR/Cas9 + overexpression	*Agrobacterium*-mediated	sgRNA + Cas9	SDN-1	No	CRISPR knockout confirmed reduced tolerance; overexpression increased chlorophyll, proline, peroxidase activity; field-level comparisons confirmed superior growth of edited lines over wild-type under salt stress	Liu T. et al. (2023)
*GmSOS1*	Plasma membrane Na+/H+ antiporter; essential for Na+ exclusion via conserved SOS pathway	CRISPR/Cas9	*Agrobacterium*-mediated	sgRNA + Cas9	SDN-1	No	Knockout resulted in elevated Na+ accumulation and disrupted ionic homeostasis, confirming essential role in salt tolerance	Yazar et al. (2025)
*GmAKT1*	Potassium channel; regulates K+ uptake and salt stress tolerance via GmCBL9-GmCIPK6 complex	CRISPR/Cas9 + overexpression	*Agrobacterium*-mediated	sgRNA + Cas9	SDN-1	No	Knockout reduced K+ content and heightened salt sensitivity; overexpression enhanced K+ uptake and tolerance mediated by GmCBL9-GmCIPK6 regulatory complex	Feng et al. (2025)
Drought stress								
*GmHdz4*	HD-Zip I transcription factor; negative regulator of drought tolerance	CRISPR/Cas9	*Agrobacterium*-mediated	sgRNA + Cas9	SDN-1	No	Knockout enhanced drought tolerance with improved root architecture, increased total root length, root surface area, osmolyte accumulation, and elevated SOD, CAT, and POD activities	Zhong et al. (2022)
*GmOFP8*	OVATE family protein; positive regulator of drought tolerance and nodulation	CRISPR/Cas9 + overexpression	*Agrobacterium*-mediated (EHA101)	sgRNA + Cas9	SDN-1	No	Knockout resulted in ∼20% survival vs. ∼70% in overexpression lines; integrates brassinosteroid signaling with drought adaptation and nitrogen fixation via GmOFP8-GmSK2 interaction	Zhang et al. (2025)
*GmPM35*	Seed maturation protein; positive regulator of drought-response signaling	Overexpression only	*Agrobacterium*-mediated	N/A	SDN-3	No	Improved photosynthesis, water content, antioxidant capacity; upregulated DREB2A, RD17, RD26, and CBF3 under water deficit	Wang et al. (2025b)
Heat stress								
*GmBSK1*	Brassinosteroid-signaling kinase; positive regulator of heat resilience	CRISPR/Cas9 + overexpression	*Agrobacterium*-mediated	sgRNA + Cas9	SDN-1	No	Knockout increased ROS accumulation and heat sensitivity; overexpression enhanced ROS scavenging and maintained yield through GmBSK1-GmGSK1-GmBES1.5 regulatory module	Hou et al. (2024)
*GmHsp90A2*	Heat shock protein 90; chaperone protein; positive regulator of thermotolerance	Overexpression only	*Agrobacterium*-mediated (EHA105)	N/A	SDN-3	No	Overexpression reduced chlorophyll degradation, electrolyte leakage, lipid peroxidation; upregulated HsfA1s, Hsp70s, GmGST, and GmCAT under 38° heat stress	Huang et al. (2019)
Heavy metal stress								
*GmAMT2.1/2.2*	Ammonium transporters; regulate NH_4_^+^-N uptake and rhizosphere microbiome assembly under Cd stress	CRISPR/Cas9 + overexpression	*Agrobacterium-*mediated (EHA105)	sgRNA + Cas9	SDN-1	No	Double knockout increased Cd sensitivity and reduced NH4+-N uptake; overexpression recruited beneficial microorganisms including *Tumebacillus* and *Penicillium*, reducing Cd accumulation, and upregulating detoxification genes	Cai et al. (2024)
*GmMYB84*	R2R3-MYB transcription factor; regulates the phenylpropanoid pathway and antioxidant defense under Cd stress	CRISPR/Cas9 + overexpression	*Agrobacterium*-mediated (EHA105)	sgRNA + Cas9	SDN-1	No	Knockout showed severe growth inhibition and elevated oxidative stress; overexpression enhanced Cd tolerance through flavonoid accumulation and cell wall reinforcement via the phenylpropanoid pathway	Gong et al. (2026)

Gene abbreviations: GmAITR, *Glycine max* ABA-induced transcription repressor; GmARM, *Glycine max* armadillo repeat protein; GmNHL1, *Glycine max* non-host resistance 1/harpin-induced 1-like 1; GmSOS1, *Glycine max* salt overly sensitive 1; GmAKT1, *Glycine max* arabidopsis K+ transporter 1; GmHdz4, *Glycine max* homeodomain-leucine zipper 4; GmOFP8, *Glycine max* OVATE family protein 8; GmPM35, *Glycine max* seed maturation protein 35; GmBSK1, *Glycine max* brassinosteroid-signaling kinase 1; GmHsp90A2, *Glycine max* heat shock protein 90 A2; GmMYB84, *Glycine max* MYB, transcription factor 84; GmAMT2.1/2.2, *Glycine max* ammonium transporter 2.1/2.2.

### 4.3 Trait-based improvement in soybean for enhanced agronomic efficiency and yield

Soybean yield is determined by a multitude of genes governing key components of ideal plant architecture, including plant height, pod number, branch number, node number, length of the internode, and grain number. This complexity is further amplified by the interaction of Genotype × Environment × Agronomic management practices (G × E × M) (Vogel et al., 2021). Genome editing strategies can therefore aid in breeding for high-value traits in soybean and, subsequently, yield. One major breeding trait is plant architecture, with stem growth habit genes forming important candidates for manipulation, particularly due to favorable attributes of semi-dwarf varieties such as lodging resistance and yield improvement. miRNAs have been identified as regulators of *SPL* genes that govern axillary bud formation and shoot branching through interaction with the homeobox protein *WUSCHEL* (WUS) (Bao et al., 2019). In their seminal study, they generated quadruple and single mutants exhibiting enhanced node numbers, thus demonstrating the utility of CRISPR/Cas9 for manipulating plant architecture in soybean.

Soybean is a facultative short-day (SD) plant with photoperiod sensitivity, and flowering time can be modulated by targeting genes governing the central circadian clock such as *LHY and CCA1*. *LHY* genes are redundantly involved in regulating endogenous *GA3* (Gibberellic acid) levels, flowering time, and yield levels (Dong et al., 2021). When the *LHY* gene was targeted using CRISPR/Cas9, it generated quadruple mutants that displayed stunted height and reduced internodal length, with significantly lower relative expression levels than the wild-type plants, suggesting the involvement of the GA pathway in *LHY-*mediated growth regulation (Cheng et al., 2019). Flowering in soybean involves the synchronized interplay of multiple genes such as *E1-E11, TOF11* and *12*, and *FT2,* which are reported to govern the transition from the vegetative to the reproductive phase, signifying floral development (Xia et al., 2021; Lu et al., 2017; Kong et al., 2018; Gong, 2020; Lu et al., 2020). In soybean, maturity loci E1, E2, E3, and E4 are chief contributors to flowering time variation, and their mutants are shown to be photoperiod-insensitive (Tsubokura et al., 2013; Xu et al., 2013). Of them, E1 functions as a key suppressor of *FT2a* and *FT5a,* which are positive regulators of flowering and maturity induction (Xia et al., 2012; Nan et al., 2014). Han et al. (2019) generated truncated *E1* gene variants through targeted editing, which in turn disinhibited *GmFT2a/5a* expression levels and resulted in an early-flowering phenotype. Four AP orthologs are found in soybean, of which *AP1* is especially required for the transition from an inflorescence to a floral meristem and is responsible for petal and sepal differentiation (Sawettalake et al., 2017; Chen et al., 2020). *Dt1* and *Dt2* are some well-elucidated genes encoding traits for Terminal Flower 1 protein and MADS-domain transcription factors, respectively. The gene *Dt2* belongs to the *APETALA (AP1)*/*SQUAMOSA* subfamily, which represses transcription and thereby modulates semi-determinate growth habit in soybean by binding to the *Dt1* promoter through its interaction with the *SUPPRESSOR OF OVEREXPRESSION OF CONSTANS 1* (*SOC1)* gene (Li et al., 2024). Using CRISPR/Cas9 technology, Chen et al. (2020) generated a quadruple *GmAP1* mutant that exhibited altered floral morphology, delayed flowering, increased internodal length, and enhanced internode numbers, generating taller plants than the wild type. Overexpression of *GmAP1a* under short-day conditions resulted in early flowering and reduced plant height. Thus, CRISPR/Cas9 is an invaluable reverse genetics tool for dissecting flowering regulatory networks in soybean molecular breeding.

Another key determinant to target for yield enhancement is traits associated with seed number and seed morphology. Seed development occurs through three distinct stages: seed set, growth, and maturity. Recent research has established critical coordination between the embryo and endosperm, where nutrient supply and transfer are mediated by sugar transporters. By targeting the *GmSWEET* transporter using CRISPR/Cas9, Wang et al. (2019a) showed its importance in sucrose transport and embryo uptake, with mutants exhibiting delayed embryo development and seed abortion. Seed weight and number of seeds per plant evidently correlate with yield, and their regulation has been a major focus of targeted editing efforts. A repressor complex consisting of *PEAPOD2,* KINASE-INDUCIBLE INTERACTING 8/9 *(KIX8/9)*, and *TOPLESS* (TPL) proteins was found to affect proliferation of the meristemoid by negatively regulating cyclin expression, as found by Baekelandt et al. (2018) in *Arabidopsis.* Nguyen et al. (2021) identified *GmKIX*8 as a negative regulator of organ size in soybean, and CRISPR/Cas9-mediated editing resulted in increased leaf and seed size attributable to enhanced cell proliferation. Considering pod morphology, Kanazashi et al. (2018) edited the *PPD* at two loci, with confirmed mutants at 33% of targeted loci exhibiting morphological abnormalities, including dome-shaped trifoliate leaves, severely twisted pods, and decreased seed production. Additionally, Walton et al. (2020) developed a nearly PAM-free SpRY variant, expanding the target range for soybean tremendously. It was employed by Chen et al. (2024) to edit *GmFAD2-1A* and *GmFAD2-1B* genes, producing high oleic acid levels in the T2 double mutant lines.

Being a leguminous crop, soybean harbors a unique symbiotic nitrogen fixation system in its roots that enhances overall plant output and seed nutritional quality (Igiehon et al., 2024). Nodulation is a cascade-dependent process involving numerous signaling components and downstream transcription factors. Super nodulation is a feature found in leguminous crops that impacts plant growth and development, where the total number of root nodules may be governed by a negative feedback mechanism known as autoregulation of nodulation (AON). This serves as a regulatory link between carbon and nitrogen balance within the host plant. Wang et al. (2019b) demonstrated the interaction of *GmNINa-miR172c-NNC1* with *GmRIC1* and *GmRIC2* genes as a master regulatory switch in regulating and balancing nodulation and AON in soybean. In a parallel study, Bai et al. (2020) generated *gmric1/gmric2* double mutants and *gmrdn1-1/1-2/1-3* triple mutant lines using CRISPR/Cas9 multiplexing, generating soybean plants with enhanced nodule numbers and decreased nodulation. The applications in agronomic trait improvement in soybean using CRISPR/Cas9 are provided in Table 7.

**TABLE 7:** Application of CRISPR/Cas9 for agronomic trait improvement in soybean.

Target gene	Gene function	CRISPR system used	Delivery method	CRISPR reagent	SDN type	Transgene-free	Research outcome	Reference
Flowering and photoperiod regulation								
*SPL family*	Plant architecture	CRISPR/Cas9	*Agrobacterium*-mediated	Cas9 + gRNA	SDN-1	Yes	Quadruple mutant and single mutant soybean plants showing increased node numbers and dry weight	Bao et al. (2019)
*GmEIL3, EIL4, EIN2L*	Flower bud differentiation and regulation of early flowering	CRISPR/Cas9 multiplexing	*Agrobacterium*-mediated	Cas9 + multiple gRNA	SDN-1	No	Triple mutant of soybean with early-flowering time and high yield phenotype	Cheng et al. (2023)
*GmE1*	Flowering/Photoperiod	CRISPR/Cas9	*Agrobacterium*-mediated	Cas9 + gRNA	SDN-1	Yes	Truncated E1 protein causes early flowering, suggesting its negative regulation of flowering	Han et al. (2019)
*GmE1*	Photoperiod sensitivity/Plant architecture	CRISPR/Cas9	*Agrobacterium*-mediated	Cas9 + gRNA	SDN-1	Yes	Mutants showed determinate stem habit, fewer branches	Wan et al. (2022)
*GmFT2a, GmFT4, GmFT5a*	Flowering	CRISPR/Cas9	*Agrobacterium*-mediated	Cas9 + gRNA	SDN-1	No	Double mutants displayed late flowering and could have tremendous yield potential	Cai et al. (2020a)
*GmFT2a, GmFT4*	Flowering	CRISPR/Cas9 base editing	*Agrobacterium*-mediated	BE (base editor) + sgRNA	SDN-1	No	Phenotypic analysis of the gene with respect to the base-editing construct	Cai et al. (2020b)
Plant architecture and growth regulation								
*SPL*	Plant architecture	CRISPR/Cas9	*Agrobacterium*-mediated	Cas9 + gRNA	SDN-1	Yes	Quadruple mutant and single mutant soybean plants showing increased node numbers and dry weight	Bao et al. (2019)
*GmLHY1, GmLHY2*	Plant architecture	CRISPR/Cas9	*Agrobacterium*-mediated	Cas9 + gRNA	SDN-1	Yes	Regulates GA-mediated pathway for plant height. Quadruple mutant soybean plant exhibiting reduced plant height and internodal length	Cheng et al. (2019)
*GmAS1/2*	Leaf architecture	CRISPR/Cas9	*Agrobacterium*-mediated	Cas9 + gRNA	SDN-1	Yes	Involvement in leaf development and polarity establishment	Xu et al. (2025)
*GmCOP1*	Photomorphogenic inhibitor	CRISPR/Cas9	*Agrobacterium-*mediated	Cas9 + gRNA	SDN-1	Yes	High accumulation of bZIP TFs in double mutants and enhanced performance under high-density conditions	Ji et al. (2022)
Floral development								
*GmAP1*	Floral organ development	CRISPR/Cas9	*Agrobacterium*-mediated	Cas9 + gRNA	SDN-1	Yes	Loss-of-function mutants showed delayed flowering, increased node number and length	Chen et al. (2020)
Seed development and yield traits								
*GmSWEET15*	Seed and embryo development	CRISPR/Cas9	*Agrobacterium*-mediated	Cas9 + gRNA	SDN-1	Yes	Delayed embryo development and abortion of seeds showed involvement of SWEET15 in embryo development	Wang et al. (2019a)
*GmKIX8*	Seed weight	CRISPR/Cas9	*Agrobacterium*-mediated	Cas9 + gRNA	SDN-1	Yes	Enhanced size in leaf and seeds due to cell proliferation	Nguyen et al. (2021)
*GmJAG1*	Number of seeds per pod	CRISPR/Cas9	*Agrobacterium*-mediated	Cas9 + gRNA	SDN-1	Cas9-free	Yield enhancement in mutants by 8.7% on average due to increased number of seeds per pod (NSPP) and total seeds per plant	Cai et al. (2021)
Pod and reproductive morphology								
*GmRDN1-1,1-2, 1-3*	Growth and nodulation in soybean	CRISPR/Cas9	*Agrobacterium*-mediated	Cas9+gRNA	SDN-1	No	Decreased nodulation was observed in a triple mutant of soybean, suggesting the role of these genes in nodulation and growth	Bai et al. (2020)
*GmPPD1, GmPPD2*	Pod morphology	CRISPR/Cas9	*Agrobacterium*-mediated	Cas9 + gRNA	SDN-1	Yes	Mutants showed deformed pod morphology	Kanazashi et al. (2018)

Gene abbreviations: SPL family, squamosa promoter binding protein-like; GmRDN1-1,1-2, 1-3, root determined nodulation; GmLHY, late elongated hypocotyl; GmFT, flower locus T; GmAP1, apetala1; GmEIL3, EIL4, and EIN2, ethylene insensitive 2, ethylene insensitive 3, ethylene insensitive 2-like; GmSWEET15, sugars will eventually be exported transporter; GmKIX8, kinase-inducible domain interacting 8; GmJAG1, jagged 1; GmPPD1, Peapod; GmAS1/2, asymmetric leaves; GmCOP1, constitutive photomorphogenic 1.

### 4.4 Genetic improvement of soybean for enhanced nutritional quality

Soybean seeds are rich repositories of high-quality protein and edible oils that provide all nine essential amino acids required for proper growth and human development. Targeted manipulation of genes governing protein content and fatty acid profiles holds considerable commercial and nutritional value for both the agricultural industry and the consumer market. However, the quality of soybean oil is compromised by high oxidative instability arising from substantially elevated levels of α-linolenic acids and reduced monounsaturated fatty acid (MUFA) content, which diminishes its competitive appeal relative to MUFA-enriched oils such as olive oil (Do et al., 2019).

In their pioneering study, Do et al. (2019) simultaneously edited two fatty acyl desaturase *FAD2 genes*, that is, *GmFAD2-1A* and *GmFAD2-1B,* using CRISPR/Cas9, where double homozygous mutant plants exhibited 60% higher oleic acid than the wild type. *FAD2* paralogs were subsequently targeted using a DNA-free CRISPR/Cpf1 system by Kim H. et al. (2017), where Cpf1 produced larger and more specific deletions at the target loci than a Cas9-based system. Ma Y. et al. (2021) edited a pair of *FATB* protein-coding genes in which single and double mutants showed significantly depleted levels of saturated fatty acids, including palmitic and stearic acid, relative to wild-type plants, thereby enhancing the overall beneficial fatty acid ratio. To enhance the levels of MUFA in soybean seeds, Li et al. (2023) targeted the phosphatidylcholine:diacylglycerol cholinephosphotransferase (*PDCT*) gene, which encodes a key enzyme that interconverts phosphatidylcholine (*PC)* and diacylglycerol *(DAG),* by simultaneously knocking out *GmPDCT1* and *GmPDCT2* using two sgRNAs*.* The resulting double mutants showed a significant increase in MUFA content, enhancing the nutritional value of soybean seed oil.

In addition to its rich oil profile, soybean seed is abundant in essential amino acids, making it a source of complete protein. Proteome rebalancing is a phenomenon adopted by the plant to maintain an intrinsic total seed protein content within a genetically defined framework that has been investigated as a target for improving seed protein levels (Herman, 2014). Shen et al. (2022) identified *ABI3-*interacting protein 2 (*AIP2*), which regulates the degradation of the abscisic acid-insensitive 3 (*ABI3*) transcription factor responsible for ABA-regulated gene expression, acting as a negative regulator of seed protein accumulation. Using CRISPR/Cas9 technology, they silenced both alleles of the *AIP2* gene, which resulted in enhanced seed protein content without any consequential impact on seed oil levels. An interesting study by Wang et al. (2025c) demonstrated that editing the *NF-YC4* promoter elevated protein levels in soybean and rice with a concomitant reduction in carbohydrate levels, thereby emphasizing the prospects of editing the cis-element to modify complex agronomic traits.

Apart from enhancing the desirable nutritional traits, consistent efforts using this technique have been made to address the unappealing characteristics in soybean that limit the consumer acceptability of soybean as a human food source. Raffinose family oligosaccharides (RFOs) are prominent antinutritional factors in seeds that cause gas and indigestion upon consumption (Salvi et al., 2022). Using the CRISPR/Cas9 technique, Le et al. (2020) knocked down galactinol synthase-encoding genes (*GmGOLS1A, GmGOLS1B)*, where double mutants showed a significant decrease in stachyose and raffinose content, and little reduction was found in verbascose content. By applying a multiplexed CRISPR/Cas9 approach, Cao et al. (2022) simultaneously knocked out the raffinose synthase *(RS)* genes, *RS2* and *RS3,* to decrease the content of RFOs in mature soybean seeds. Phytic acid (inositol 1,2,3,4,5,6- hexaphosphate) is another key anti-nutrient which complexes with iron and zinc, hindering their bioavailability to humans. Song et al. (2022) targeted *GmIPK*1, which encodes the enzyme responsible for converting inositol 1,3,4,5,6-pentaphosphate to inositol 1,2,3,4,5,6-hexaphosphate, using CRISPR/Cas9 and observed reduced phytic acid levels in edited lines without adverse effects on seed development or growth.

Off-flavor or beany flavor is a major quality limitation that restricts human consumption of soybean products. Wang et al. (2020) targeted three genes in the lipoxygenase (*LOX)* family, that is, *LOX1, LOX2,* and *LOX3,* which are typically responsible for the enzymatic reactions that generate beany flavor compounds. Knocking out these genes using the CRISPR/Cas9 technique reduced off-flavor and improved palatability, thus resulting in the generation of lipoxygenase-free soybean mutant lines. These collective studies demonstrated that CRISPR/Cas9-based genome editing enables precise manipulation of key metabolic pathways governing seed composition, and this technology holds significant promise for developing soybean varieties with improved nutritional quality and enhanced market value. With the data presented in Table 8, the applications of CRISPR/Cas are well elucidated in the improvement of nutritional factors in soybean.

**TABLE 8:** Application of CRISPR/Cas9 for nutritional improvement in soybean.

Target gene	Gene function	CRISPR system used	Delivery method	CRISPR reagent	SDN type	Transgene-free	Research outcome	Reference
Oil quality								
*GmFAD2*	Oil quality	CRISPR/Cas9	*Agrobacterium*-mediated	Cas9 + gRNA	SDN-1	Yes	High oleic acid and low linoleic acid content in mutant soybean seeds	Do et al. (2019)
*GmFATB1*	Oil profile	CRISPR/Cas9	*Agrobacterium*-mediated	Cas9 + gRNA	SDN-1	No	Reduction in saturated fatty acids was observed with relative increase in linoleic acid	Ma J. et al. (2021)
*Gly m Bd 30K*	Oil body associated protein	CRISPR/Cas9	Biolistic transformation	SpCas9+gRNA	SDN-1	Yes	Inheritable mutation in soybean somatic embryos and transgene-free progeny	Adachi et al. (2021)
*GmSFAR4*	Oil content	CRISPR/Cas9	*Agrobacterium*-mediated	Cas9 + gRNA	SDN-1	No	Enhanced seed oil content by ∼8% in double mutants compared to the wild type	Liao et al. (2025)
*FAD2*	Oil quality	CRISPR/Cpf1	RNP-mediated protoplast culture	Cpf1 + RNP	SDN-1	Yes	Increase in oleic acid using Cpf1-RNP system with larger deletions in the target sites	Kim H. et al. (2017)
Nutritional quality								
*GmGOLS1A, GmGOLS1B*	Nutritional quality	CRISPR/Cas9	*Agrobacterium*-mediated	Cas9+gRNA	SDN-1	Yes	Lowered levels of RFOs in soybean seeds	Le et al. (2020)
*IPK1*	Nutritional quality	CRISPR/Cas9	*Agrobacterium*-mediated	Cas9 + gRNA	SDN-1	No	Reduced phytic acid content in seeds	Song et al. (2022)
*GmNF-YC*4	Nutritional quality	CRISPR/Cas9	*Agrobacterium*-mediated	Cas9 + gRNA	SDN-1	Yes	Increase in seed and leaf protein content	Wang et al. (2025c)
*RS2, RS3*	Nutritional quality	CRISPR/Cas9	*Agrobacterium*-mediated	Cas9 + multiple gRNA	SDN-1	No	Reduction in levels of RFOs	Cao et al. (2022)
Allergens								
*LincCG1*	Allergen	CRISPR/Cas9	*Agrobacterium*-mediated	Cas9 + gRNA	SDN-1	Yes	Reduced conglycin content	Song et al. (2024)
*GmBbi*	Allergen	CRISPR/Cas9	*Agrobacterium*-mediated	Cas9 + gRNA	SDN-1	No	Lowered protease inhibitor content	Kim et al. (2024)
Seed protein content and flavor								
*LOX1, LOX2, LOX3*	Flavor of seeds	CRISPR/Cas9	*Agrobacterium*-mediated	Cas9 + gRNA	SDN-1	Yes	Reduction of beany flavor in transgene soybean lipoxygenase-free soybean lines	Wang et al. (2020)
*AIP2a, AIP2b*	Seed protein content	CRISPR/Cas9	*Agrobacterium*-mediated	Cas9 + gRNA	SDN-1	No	Gene inactivation resulted in enhanced seed protein content	Shen et al. (2022)
Fatty acid content								
*GmFATA1, GmFATA2*	Fatty acid content	CRISPR/Cas9	*Agrobacterium*-mediated	Cas9 + gRNA	SDN-1	No	Growth defect in mutants, lower triglycerol abundance at early seedling stage	Liao et al. (2024)
*GmPDCT*	Fatty acid content in seeds	CRISPR/Cas9	*Agrobacterium*-mediated	Cas9 + gRNA	SDN-1	No	Generation of soybean seeds with enhanced MUFA by maneuvering seed oil composition	Li et al. (2023)
*GmPDCT*	Fatty acid content in seeds	CRISPR/Cas9	*Agrobacterium*-mediated	Cas9 + gRNA	SDN-1	No	Generation of soybean seeds with enhanced MUFA by maneuvering seed oil composition	Li et al. (2023)
Astringent taste								
*GmBAS1* and *GmBAS2*	Astringent taste	CRISPR/Cas9	Particle bombardment	SpCas9+gRNA	SDN-1	Yes	Reduction of soysaponin content in the mutants	Asa et al. (2025)

Gene abbreviations: GmFAD2, fatty acid desaturase 2; GmFATB1, fatty acyl carrier protein thioesterase; GmGOLS1A, GmGOLS1B, Galactinol synthase; GmPDCT, phosphatidylcholine:diacylglycerol cholinephosphotransferase; GmNF-YC4, nuclear factor Y; FAD2, fatty acid desaturase; RS2, raffinose synthase; IPK1, Inositol 1,3,4,5,6-pentakisphosphate 2-kinase; LOX, lipoxygenase; AIP: ABI3-interacting protein 2; lincCG1, long intergenic noncoding RNAs; GmFATA1, acyl–acyl carrier protein (ACP) thioesterases; GmBbi, Bowman–Birk protease inhibitors; GmSFAR4, seed fatty acid reducers; GmBAS, beta-amyrin synthase 1.

## 5 Multiplex gene editing in soybean

The soybean genome is characterized by its paleopolyploid origin, extensive gene duplication, subsequent diversification, gene loss, and numerous chromosomal rearrangements. Approximately 75% of the genes exist in multiple copies, rendering the genome highly redundant and complicating single-gene targeting due to potential functional compensation by homologous paralogs (Schmutz et al., 2010). To overcome this complexity, multiplexing strategies have helped to simultaneously edit multiple loci, thereby enhancing the efficiency of functional knockouts. Several advanced cloning and assembly platforms have been instrumental in constructing multi-sgRNA vectors such as Golden Gate cloning and Gateway cloning. The Golden Gate assembly exploits type II-S restriction enzymes (*BsaI*, *BbsI*) to generate non-palindromic sticky ends, thus enabling seamless multi-fragment DNA assembly and accelerating complex construct design for soybean genome engineering (Abdelrahman et al., 2021). Gateway cloning relies on site-specific recombination between attB and attP sites mediated by BP/LR clonase enzymes. It has been widely employed to assemble multiple sgRNA expression cassettes into a single binary. However, this process introduces 25 bp “scar” sequences flanking the final insert, which might occasionally affect transcriptional efficiency (Luo et al., 2018).

Two principal strategies facilitate the cloning of multiple gRNAs into a single delivery construct: the more commonly used two-component transcriptional unit (TCTU), and a simple version, the single transcriptional unit system (STU). The conventional TCTU system relies on the independent expression of Cas9 and sgRNAs under separate promoters. In this configuration, Cas9 is typically driven by an RNA polymerase (Pol) II promoter, while individual sgRNAs are each expressed from dedicated Pol III promoters such as the U6 or U3 small nuclear RNA gene promoters (Zhang et al., 2016). In contrast, the STU system uses a more compact structure in which the Cas9 is fused with an endoribonuclease and multiple sgRNAs are co-expressed from a single Pol II promoter within a unified transcriptional cassette. Carrijo et al. (2021) employed these two strategies, in which TCTU systems use a *GmUbQ* promoter to drive Cas9 expression and a *GmU6* promoter to independently drive multiple sgRNA transcripts. The STU configuration placed the fused Cas9 and gRNA cassettes under a single *GmUbQ* promoter. The STU system accommodated more than four sgRNAs in multiplex designs targeting two different genes, *GmIPK1* and *GmIPK2,* both of which encode the phytic acid biosynthesis pathway. The STU system offers a practical advantage in its reduced construct size (smaller by 1000 bp), which simplifies the assembly and confers greater flexibility for accommodating additional sgRNA units. However, they generally exhibit slightly lower editing efficiency relative to the more complex and higher-performing TCTU platform. Together, these two systems could be robust platforms for multiplexed editing, where STU systems offer speed and simplicity for rapid multi-gene targeting, while TCTU systems provide the higher editing efficiencies.

An alternative strategy involves designing polycistronic mRNA transcripts in which each individual sgRNA is separated by cleavage sites. These include 20-bp hairpin sequences derived from Csy4 (a CRISPR-associated RNA-based endonuclease from *Pseudomonas aeruginosa*) or 77-bp tRNA sequences that leverage the endogenous processing machinery of plants. Another class is the self-processing ribozyme, which enables the transcription and generation of functional, individual sgRNA units from a single transcript without the need for cellular enzymes (Tsai et al., 2014; Xie et al., 2015; Gao and Zhao, 2014; Tang et al., 2016). These multiplex cloning systems provide robust platforms for soybean molecular breeding programs. A comparative summary between the two systems has been documented in detail in Table 9.

**TABLE 9:** Integrated summary of multiplex gene editing in soybean.

Trait modified	Editing strategy	Target genes	Objective	Reference
Nutritional quality	TCTU, STU	*Raffinose synthase* (*RS2* and *RS3*)	Reduce anti-nutritional RFOs for better digestibility and food/feed quality	Cao et al. (2022)
Biotic stress	TCTU	*Isoflavone synthase (IFS), flavone synthase (FNS)*, and *flavanone-3-hydroxylase (F3H)*	More isoflavone, resistance to soybean mosaic virus	Zhang et al. (2020)
Flowering time and pod set	Golden gate	*Ethylene insensitive 2 (EIN2)* and *ethylene insensitive 3 (EIN3)*	Early phenotype and high yield phenotype	Cheng et al. (2023)
Antinutritional factor	TCTU, STU	*Inositol-pentakisphosphate 2-kinase (IPK1* and *IPK2)*	Reduction of phytic acid	Carrijo et al. (2021)
Antinutritional factor	Golden gate assembly	*Raffinose synthase (RS2, RS3*, and *RS4) Stachyose synthase (STS)*	Reduce anti-nutritional RFOs for better digestibility	Lin et al. (2023)
Fatty acid pool	STU and polycistronic-tRNA-gRNA strategy	*Fatty acid desaturase 2 (FAD2)* and *fatty acyl-ACP thioesterase B (FATB)*	Modifying fatty acid pool	Kim et al. (2021)

## 6 Transgene-free genome editing techniques in soybean

Recent innovations in DNA-free CRISPR delivery systems have successfully circumvented inherent constraints of conventional transformation-based methods. While the classical *Agrobacterium-*mediated stable transformation is foundational, it invariably results in the genomic integration of CRISPR/Cas9 constructs, Cas9, and selectable marker genes. This integration not only raises regulatory scrutiny but also necessitates laborious generational segregation to eliminate transgenes, thereby perpetuating the “GMO” stigma that hinders market adoption and impedes public acceptance. The genotype-dependence of soybean transformation makes it imperative to adopt transgene-free approaches (Teng, 2024).

Transgene-free genome editing has emerged as a transformative approach, enabling precise genetic modifications without the stable integration of foreign DNA. A critical scientific rationale for transgene-free editing is the reduction of unintended mutagenesis, where continual expression of gene editing components intensifies off-target effects. Therefore, genome editing without permanent insertion of foreign DNA will have minimal risk of unwanted off-target mutations (Bhattacharjee et al., 2023). The final edited plants tend to be free of foreign DNA and are genetically indistinguishable from naturally occurring mutants, thereby qualifying as non-GMO plants. This distinction confers lighter regulatory oversight, faster commercialization, and better public acceptance. The principal strategies include segregation-based removal of transgenes, transient expression systems, direct delivery of Cas9-ribonucleoprotein complexes (RNPs), and *in-planta* particle bombardment of RNPs (iPB-RNP). The foundational segregation-based approach introduces a Cas9 expression cassette, guide RNA sequences, and selectable markers into plant cells using *Agrobacterium tumefaciens*-mediated transformation or biolistic delivery, followed by systematic elimination of the transgenes through Mendelian segregation across successive generations. Transient expression systems provide an alternative route for plant tissues, where CRISPR/Cas9 RNPs are directly delivered through particle bombardment or *Agrobacterium*-mediated infiltration without stable genomic integration, bypassing the need for antibiotic selection (Zhang et al., 2021).

A conceptually distinct strategy employs an engineered plant negative-strand RNA virus from Sonchus Yellow Net rhabdovirus (SYNV), which exploits its non-integrative biology to deliver CRISPR/Cas9 cassettes *in planta*, achieving editing efficiencies of up to 90% for single-gene and multiplex targets (Ma et al., 2020). Successful demonstrations have been applied in soybean; Subburaj et al. (2022) employed PEG-mediated delivery of Cas9-RNPs to edit *GmCPR5,* generating InDel mutations with efficiencies ranging from 4.2% to 18.1% and producing frameshift mutations. Extending this methodology, Kuwabara et al. (2024) introduced the SAM stem cells-based iPB-RNP system, which eliminates transgene integration and enables direct regeneration of edited plants (E0) from treated embryos, surpassing the laborious need for tissue culture. A proportion of E0 plants exhibited chimeric or bi-allelic mutations, while the subsequent generations yielded stable, non-chimeric mutants. These complementary strategies highlight a clear transition toward regeneration-independent and genotype-flexible editing systems in soybean. Importantly, the transient nature of RNP activity minimizes off-target effects while aligning with evolving regulatory frameworks. With continued refinement, transgene-free genome editing is poised to accelerate the development of soybean cultivars with favorable traits.

## 7 Regulatory framework for genome-edited plants in the global scenario

The regulatory landscape for genome-edited crops is marked by significant jurisdictional differences. Many nations have exempted specific categories of genome-edited plants from existing genetically modified organism (GMO) regulations, particularly those involving site-directed nucleases type 1 (SDN-1) and type 2 (SDN-2). These techniques generate targeted deletions or allelic modifications of endogenous genes without stable integration of foreign DNA. The scientific basis for these exemptions depends on the principle that SDN-1 introduces a double-stranded break that is repaired through non-homologous end joining, resulting in mutations indistinguishable from those arising through natural or conventional mutagenesis. Additionally, the editing machinery functions in a transient manner and is not stably integrated into the genome of the final commercial product, as no exogenous, recombinant, or foreign genetic material remains heritable. There is no introduction of novel gene recombinations with the species (Entine et al., 2021).

SDN-2 introduces precise allelic variants using short donor templates that could theoretically exist within the natural gene pool of the species. The repair template is a short oligonucleotide that introduces a specific nucleotide substitution. As the template itself is not integrated or heritably transmitted, it does not leave foreign DNA in the final product. The changes occurring through SDN-2 are equivalent to SNPs or small sequence replacements that already exist within the natural allelic diversity of the species or could arise naturally. Under the product-based regulation followed by the United States, Canada, Argentina, and many Asian countries, the process becomes irrelevant, as it considers only the molecular characteristics of the final product (Whelan and Lema, 2019). In contrast, SDN-3 involves inserting foreign DNA templates repaired via homology-directed repair (HDR), thereby introducing exogenous genetic material at a defined locus and is consequently regulated at par with transgenics (Prasad and Chimata, 2023).

The regulatory frameworks that are followed globally differ primarily in their legal “triggers” and process-based regulation. In the United States, Canada, and numerous South American countries, the regulations were harmonized early and widely, where the frameworks treated SDN-1 and SDN-2 goods similar to conventionally grown genotypes (Schmidt et al., 2020). They adopted a progressive product-based approach that exempts products lacking stably integrated foreign DNA from GMO regulations (Whelan and Lema, 2019). In a similar fashion, Ecuador explicitly omitted SDN-1 products from genetically modified organism (GMO)-based regulations (Entine et al., 2021). The regulatory approach in Canada is based on product novelty rather than the process used to create it, whereas the USDA in the United States adopted a voluntary consultation process that generally exempted single deletions and single base-pair substitutions (Kalaitzandonakes et al., 2023). In Africa, National Biosafety Management Agency (2020) and National Biosafety Authority (2022) adopted the emerging case-by-case approach, which permits exemption from rigorous pre-market verification on the absence of foreign DNA, which aligns well with the African Union Agenda 2063. The restrictive process-based approach followed by the European Union (EU) and New Zealand classifies all products produced through genome editing under GMO regulations, irrespective of whether foreign DNA persists in the final product (Buchholzer and Frommer, 2023). However, reforms are under discussion; they have reconsidered their stance and proposed new regulations for plants made using specific new genomic techniques after taking into account discussions in the public forum and scientific community (European Parliament, 2021; European Commission, 2022). Norway amended its Gene Technology Act to loosen restrictions on modified crops (Kjeldaas et al., 2021). In March 2022, the Swiss parliament resolved to remove transgene-free altered lines from genetic engineering laws. In 2019, Russia legislated that crops altered without trans genes will be regarded on par with traditional breeds (Dobrovidova, 2019). Many countries in Asia had a forward-thinking approach with respect to subjecting the regulations around the genome-edited organisms, especially crops. Japan was an early adopter that treated genome editing and traditional breeding in the same regulatory purview (Normile, 2019); China released draft guidelines in 2022 exempting SDN-1/2 plants devoid of exogenous DNA. In May 2022, the Philippines excluded genome-edited plants lacking genetic material combinations from GMO regulation (USDA Report CH2022-026). One of the notable agreements between Bangladesh, Nepal, Sri Lanka, and Cambodia was “Seeds without borders” to promote regional harmonization in South Asia (Gauchan et al., 2020). The regulatory frameworks for the global scenario have been depicted in Table 10.

**TABLE 10:** Overview of regulatory policies for genome-edited crops in major countries.

Region/Country	Status	Process-/product-based evaluation	SDN-1	SDN-2	SDN-3/cisgenic	Transgenic
South America						
Argentina	Final	Product	Not GMO (if no foreign DNA and no novel genetic combination)	Not GMO (if no foreign DNA and no novel genetic combination)	Not GMO (perfect allelic replacement)	GMO
Chile	Final	Product	Not GMO (no foreign DNA)	Not GMO (no foreign DNA)	Not GMO (no foreign DNA in product)	GMO
Brazil	Final	Case by case	Not GMO (if no recombinant DNA/RNA)	Not GMO (if no recombinant DNA/RNA)	Not GMO (if no recombinant DNA/RNA)	GMO
Colombia	Final	Product	Not GMO (absence of foreign DNA)	Not GMO (absence of foreign DNA)	Not GMO (absence of foreign DNA)	GMO
Honduras	Final	Product	Not GMO (no foreign DNA)	Not GMO (no foreign DNA)	GMO-like	GMO
Paraguay	Final	Product	Not GMO (no foreign DNA)	Not GMO (no foreign DNA)	GMO	GMO
North America						
Canada	Final	Product	Not automatically GMO (assessed based on novelty)	Not automatically GMO (assessed based on novelty)	GMO	GMO
United States (USDA/crops)	Final	Product	Not GMO (absence of foreign DNA)	Not GMO (absence of foreign DNA)	GMO (case by case)	Depends
Asia and Oceania						
China	Draft/In progress	Process	Not GMO (if no exogenous DNA)	Not GMO/GMO (if no exogenous DNA/foreign source of the template)	GMO	GMO
India	Final (Memo)	Product	Not GMO (absence of foreign DNA)	Not GMO (no foreign DNA)	GMO	GMO
Philippines	Final	Product	Not GMO (if no foreign DNA)	GMO/Not GMO (case by case)	Not GMO	GMO
Japan	Final	Product	Not GMO (absence of foreign DNA)	Case-by-case (Non-GMO if the template DNA is from the same or a sexually compatible species)	GMO	GMO
Australia	Final	Product	Not GMO (if no foreign DNA)	GMO	GMO	GMO
New Zealand	Current legislation	Process	GMO	GMO	GMO	GMO
Africa						
Nigeria	Final	Product	Not GMO (if no new genetic combination)	Not GMO (if no new genetic combination)	GMO	GMO
Kenya	Final	Product	Not GMO (if no new genetic combination)	Not GMO (if no new genetic combination)	GMO	GMO
Europe and Middle East						
European Union	ECJ Decision (2018)	Process	GMO	GMO	GMO	GMO
Norway	Proposed	Not available	Not GMO (if no foreign DNA)	GMO	GMO	GMO
Switzerland	In progress	Not available	Not GMO (if transgene-free)	Not GMO (if transgene-free)	GMO	GMO
United Kingdom	In progress (expected June 2022)	Product	Not GMO (exempt)	Not GMO (exempt)	GMO	GMO
Russia	Final (Decree)	Product	Not GMO (transgene-free)	Not GMO (transgene-free)	GMO	GMO
Israel	Final	Product	Not GMO (absence of foreign DNA from a sexually incompatible source)	Not GMO (absence of foreign DNA from a sexually incompatible source)	Not GMO (absence of foreign DNA from a sexually incompatible source)	GMO

References: Entine et al. (2021), Whelan and Lema (2019), Schmidt et al. (2020), Gatica-Arias (2020), USDA-APHIS (2020), and Buchholzer and Frommer (2023); For updated country-level regulatory processes, see Convention on Biological Diversity (https://bch.cbd.int/protocol/parties).

### 7.1 Regulation of genome editing organisms in India

India established its regulatory framework that governs the regulation of genetically engineered organisms and their by-products under the “Rules for the Manufacture/Use/Import/Export and Storage of Hazardous Microorganisms, Genetically Engineered Organisms or Cells,” 1989 (Rules 1989), notified under the Environment (Protection) Act, 1986. This has enlisted six competent authorities, namely, the Institutional Biosafety Committee (IBSC), the Review Committee on Genetic Manipulation (RCGM), the Genetic Engineering Appraisal Committee (GEAC), the Recombinant DNA Advisory Committee (RDAC), the State Biotechnology Co-Ordination Committee (SBCC), and the District Level Committee (DLC), to ensure biosafety oversight. In a historic move made in 2022, with the promulgation of “Guidelines for the Safety Assessment of Genome Edited Plants,” it explicitly exempted SDN-1 and SDN-2 products from regulation under Rules 7-11, granted under Rule 20 by the Ministry of Environment, Forests, and Climate Change, Govt. of India. These edits are treated as conventional crops, while SDN-3 products, which involve foreign DNA templates, remain regulated under the GMO provisions of Rules 1989 (Prasad and Chimata, 2023) (Figure 3). The recent breakthrough development of gene-edited rice varieties in India, such as DRR Dhan 100 or Kamala, using CRISPR-Cas12/Cas9 and Pusa DST Rice 1 from MTU 1010 using CRISPR/Cas9 technology, has raised a beacon of hope toward the development of sustainable agriculture using advanced technologies and providing the country a winning edge amongst the global players in agriculture, as reported by the Economic Times (https://m.economictimes.com/small-) (The Economic Times, 2026).

**FIGURE 3:**
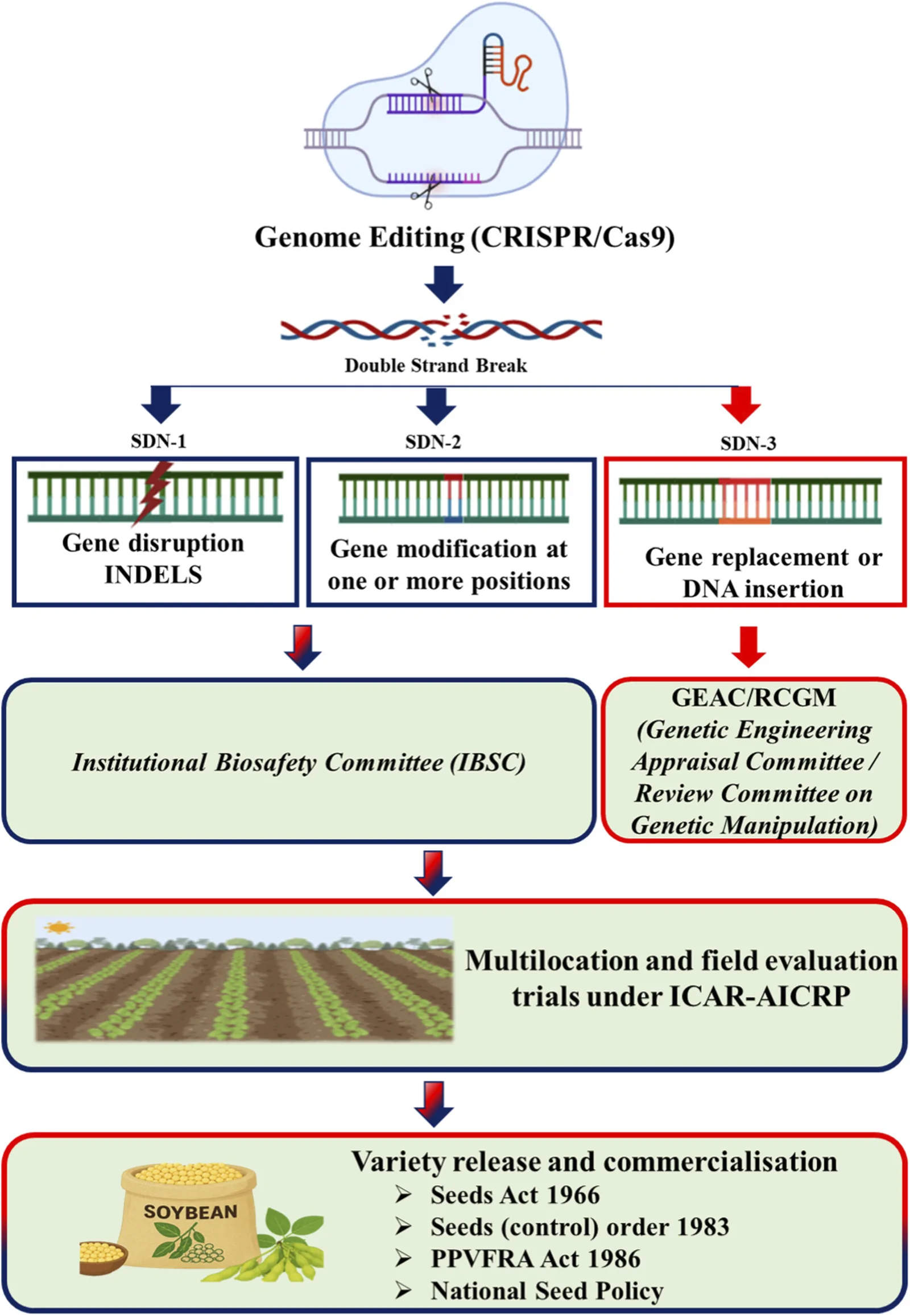
Regulatory framework for release of genetically edited crops in India: The regulatory framework for genome-edited crops is classified into three categories: SDN1, SDN2, and SDN3. The institutions responsible for the release in India have been depicted. Made in BioRender Free trial period. https://app.biorender.com/illustrations/66cf6cabf6df30a07fce25a8.

### 7.2 Status of commercially approved genome-edited soybean

The most notable precedent for commercialization is the high-oleic soybean oil developed by Calyxt (now Cibus) in the United States, produced through TALEN-mediated editing of the *FAD2* gene. This product established early regulatory precedent for genome-edited oilseed crops under the USDA product-based framework. CRISPR/Cas9-edited soybeans with elevated oleic acid content have subsequently undergone field trials in the United States with favorable outcomes (Do et al., 2019). Similarly, the USDA APHIS granted regulatory exemption to Amfora, Inc., for its CRISPR-edited ultra-high protein soybean under 7 CFR Part 340, enabling direct commercialization without the requirement of additional regulatory review (https://www.isaaa.org/kc/cropbiotechupdate/ged/article/default.asp?ID=19979).

A functionally analogous regulatory instrument has been operationalized in Brazil through Resolution No. 16 (RN16), which establishes the non-transgenic classification of genome-edited organisms as the threshold criterion for exemption from GMO regulation (da Cunha et al., 2025). By pursuing this framework, Brazil has classified Embrapa Soja’s CRISPR-edited drought-tolerant soybean as non-GMO under RN16 on the basis that the editing was carried out without the insertion of an exogenous sequence (https://www.gmwatch.org/en/106-news/latest-news/20203). This evolving international consensus across several jurisdictions represents a departure from precautionary, process-triggered frameworks that had previously imposed substantial bottlenecks on the translation of CRISPR-based soybean innovations. Harmonizing the efforts for releasing genome-edited soybean across several countries may increasingly serve as a facilitating mechanism for global market access.

### 7.3 Public perception, acceptance, and governance of genome-edited soybean

In India, soybean is a major oilseed crop, yet the country remains highly dependent on imports to support its edible demand that continues to outpace local production. Meeting oilseed productivity remains a strategic policy concern (Prashnani et al., 2024). In contrast, the relatively deregulated stance of genetically modified organisms (GMOs) in the United States has enabled GM soybean to account for more than 94% of US soy production, positioning it as a global leader in soybean oil output. Beyond the demands of food, surging interest in biofuel blending mandates is forecast to increase soybean oil use in biofuel production by 17% in 2026–27 (https://biomassmagazine.com/articles/usda-predicts-17-boost-in-soybean-oil-use-in-biofuel-production). Despite this adoption and a broad scientific consensus on their safety, societal trust in biotechnology has declined over recent years, indicating significant public concern over the safety and ethics of genetically engineered foods. This heightened scrutiny is driven by numerous factors, including social discourse and media influence, and was significantly compounded by concerted efforts of anti-biotechnology organizations that penetrate the public consciousness, polarizing their opinions through slick digital campaigns to sustain mistrust and spread dissenting views at a global level (Funk et al., 2015; Folta, 2025). Therefore, the public perception of genome-edited soybean should be approached as a governance and technological issue. A consumer survey examining attitudes toward genome-edited cold-resistant soybean reflects a mixed but cautiously optimistic outlook, with acceptance largely shaped by perceived benefits, risks, and the prevailing regulatory context (Bearth et al., 2024). The same study found that trust was among the strongest predictors of acceptance. For example, informed consumers in the United States support strongly due to their high trust in the USDA and university scientists. Japan also shows rising acceptance with knowledge gains, and South Korea is increasingly reporting purchase willingness for purchasing genome-edited (GE) products, rather than domestic cultivation. The precautionary approaches by the EU heighten risk perceptions and often polarize the audience in a skewed manner.

Most importantly, acceptance tends to be higher in regions with supportive regulatory frameworks and prior exposure to biotechnology. Therefore, it becomes the duty of science-based NGOs and media literacy to use expert-led opinions to enhance legitimacy for genome-edited soybean commercialization (Oh and Lee, 2025). A balanced Indian perspective should acknowledge both the promise and concerns around the release of genome-edited soybean. A credible middle path could be transparent risk assessment, trait-specific evaluation, post-release monitoring, and evidence of safety. The regulatory clarity paired with public-approved transparency should be communicated in consumer-centric languages, extended to farmer groups about the cultivation practices, and not only limited to expert committees so that acceptance will be higher in the entire ecosystem for genome-edited soybean.

## 8 Toward the next generation of genome editing in soybean

Over the past decade, the generation of plants with desirable traits has ushered in a transformative era driven by rapid advances in genome editing technologies. Because of the laborious and time-intensive nature of traditional breeding methods, they are increasingly supplemented by genome editing tools that enable precise targeting, easy operability, and rapid engineering of plant genomes. These technologies have become quite lucrative amidst the urgent scenario for feeding a rapidly burgeoning human population and guaranteeing food security (Wada et al., 2020). Among the most significant advanced techniques are base editors and prime editors, protein engineering, and the use of innovative delivery systems such as nanoparticles.

Base editing (BE) is an advanced technique developed using dCas9 proteins fused with DNA deaminase, which enables targeted modification of the target DNA sequence without creating double-stranded breaks (DSBs). Two principal variants of this technique are used: cytidine deaminase-incorporating DNA base editors (CBEs) and adenine deaminase-based DNA editors (ABEs). CBEs mediate the deamination of cytosine to uracil, which is subsequently converted to thymidine, resulting in the conversion of C-to-T. On the other hand, ABEs utilize adenine DNA deaminase to catalyze the deamination of adenosine to inosine, which base pairs with cytidine, resulting in the incorporation of G in the newly synthesized daughter strand (Wada et al., 2020). Wei et al. (2023) targeted and modified the P197 codon using a base editor to generate herbicide-resistant soybean germplasm by acting on the key enzyme, acetohydroxy acid synthase (AHAS), which is involved in the synthesis of branched-chain amino acids. In another study, Cai et al. (2020a) performed base editing using a modified CRISPR/nCas9 and targeted the flowering locus T *(FLT)* genes to evaluate the feasibility and efficacy of the base-editing vector. Their construct combined Cas9n (D10) nickase, rat cytosine deaminase (APOBEC1), and uracil glycosylase inhibitor (UGI) into a functional BE. Similarly, Jeong et al. (2025) identified two high-efficiency variants of ABE (ABE8e; A3A/Y130F-V04) and a CBE variant that demonstrated robust A-to-G and C-to-T base editing, respectively, in soybean protoplasts. As improvements in base-editing efficiency, target scope, and delivery systems continue to advance, base editing is expected to play an increasingly important role in accelerating trait development and enhancing the e precision of soybean breeding strategies.

Prime editing (PE) is another precise technique that avoids DSBs in the genome, thereby minimizing unintended permanent alterations in the target organism. This technique employs a fused complex of Cas9 nickase with reverse transcriptase, guided by a specially designed prime editing guide RNA (pegRNA). The engineered pegRNA comprises the desired edited sequence, a primer binding site, and a target-recognizing spacer sequence. The pegRNA drives the Cas9 nickase to the target DNA, where it nicks the PAM-containing strand. With the help of reverse transcriptase fused to the Cas9 nickase, the reverse transcription of the desired altered sequence on the pegRNA takes place after the 3′end of the nicked DNA strand hybridizes to the PBS of the pegRNA. When the reverse transcription product and the target DNA hybridize, a 3′flap with edited sequence or a 5′flap with unedited sequence is produced. While the 3′ flap is ligated to the DNA strand, the 5′ flap is cleaved by the endonuclease. DNA repair process incorporates the repair into the heteroduplex DNA, leading to stable incorporation of edited sequence into the genome (Wada et al., 2020). This method produces fewer off-target effects than conventional Cas9 because it requires two sequential hybridization steps rather than the single target-DNA-gRNA hybridization of standard Cas9. While PE has been relatively well explored in monocots, its application remains an active area of investigation. Bai et al. (2025) developed a flanking-nicks prime editing (FLICK-PE) system that generates nicks flanking the target site, and they engineered glyphosate resistance in soybean with a 2.2-fold improvement in on-target editing efficiency over Prime Editing version 3.

Another notable addition to the genome editing toolkit is transposase-assisted target site integration (TATSI), which integrates DNA fragments into specific target sites using transposable elements with enhanced efficiency and precision. Liu et al. (2024) fused a rice transposase protein with a CRISPR gRNA to enable sequence-specific targeting and excision of custom DNA within the soybean genome. This approach substantially expands the repertoire of the available genome editing tools by combining transposable element-mediated integration with CRISPR-guided site specificity.

The integration of artificial intelligence (AI) with CRISPR/Cas9 represents a further frontier, leveraging machine learning for guide RNA design, off-target prediction, and variant optimization, thereby addressing persistent limitations of accuracy and delivery. Machine learning (ML)-based algorithms trained on experimental datasets have demonstrated broad applicability across plant science disciplines, including plant breeding, stress biology, and system biology (Das et al., 2023). Next-generation resistance breeding is also anticipated to benefit from quantum computing, which offers numerous opportunities for developing climate-resilient crop varieties (Kamran et al., 2026). In a recent development, Syngenta partnered with QuantumBaseI to apply quantum computing for accelerating agricultural crop protection discovery, enhance climate resilience and enable sustainable farming (https://www.businesswire.com/news/home/20260316646014/en/Syngenta-deepens-research-capabilities-with-QuantumBasel-partnership). Wang et al. (2025d) combined AlphaFold-based protein structure prediction with CRISPR/Cas9-mediated editing of C-terminal variants of *GmSWEET10b*, demonstrating improvements in oil content in edited plants, and the engineering of novel haplotypes with optimized protein function with implications for future crop breeding.

In a parallel development, CRISPR-GPT, an AI-based large language model tool for automated guide RNA design, has been shown to substantially expedite experimental design and expand the range of options available to the researchers (Qu et al., 2025). Although this is currently validated in human adenocarcinoma cell line knockouts, the underlying framework is readily adaptable to plant systems. Deep learning-based platforms such as DeepCRISPR, DeepHF, and CRISPR-Net can predict gRNA efficiency and specificity by evaluating sequence characteristics, genomic context, and chromatin accessibility. AI algorithms can help to predict editing outcomes, repair pathway preferences, and base-editing efficiencies, thereby allowing researchers to design more effective editing strategies before laboratory validation. Machine learning models such as random forests, support vector machines, gradient boosting, and deep neural networks can predict breeding values and complex phenotypes from genomic data with higher accuracy than many traditional statistical approaches. These predictive capabilities enable breeders to identify superior genotypes and prioritize genome editing targets more efficiently, thus shortening breeding cycles (Saini et al., 2025).

Beyond these applications, researchers have recently developed plant tRNA large language models that help to identify both canonical and noncanonical tRNAs, uncovering thousands of tRNAs missed by traditional algorithms and expanding the repertoire for plant genome editing. This AI-guided mining framework provides a robust platform for advanced plant genome engineering and has been experimentally validated for multiplexed editing of genomic loci in rice and soybean (He et al., 2026). These scalable AI-guided solutions can facilitate easy designing of gene-editing experiments, curating protocols, and analyzing results, which could collectively power the future of CRISPR-developed climate-resilient crops.

## 9 Perspectives

The transgenic approaches have greatly enabled the introduction of foreign genes into crops such as soybean, expanding the genetic variability for crop improvement. However, the stiff regulatory stances and biosafety concerns have considerably increased the cost and time required for varietal development. In contrast, genome editing technologies offer a precise and predictable alternative, allowing breeders to directly modify elite cultivars within a shorter timeframe. A defining feature of genome editing is the indistinguishable nature of the resulting mutations compared to those generated through conventional mutagenesis, which can help address public and regulatory concerns commonly associated with transgenic crops.

Despite the promise, applying genome editing in soybean faces several critical challenges. Several technical constraints, such as limited control over precise edits, inefficiency of delivery of reagent systems, low regeneration efficiency, and off-target effects, continue to hinder effective transformation. The *in silico*-based prediction tools, use of morphogenic regulators, and development of in-plant or protoplast-based transformation can help mitigate these challenging problems to a considerable extent. Additionally, AI-based tools hold tremendous potential for target identification, streamlining construct design, and retrieving meaningful insights from limited available resources. The current efforts remain focused on SDN-1- and SDN-2-based approaches for gene knockout, as limited mechanistic understanding and regulatory uncertainties surrounding HDR-based repair have constrained the development of SDN-3-based precise edits. Emerging tools such as BE and PE offer significant potential to overcome these constraints. However, their application in soybean remains at an early stage and requires dedicated optimization efforts. The translation of these techniques into relevant outcomes will require dedicated germplasm-specific optimization and transformation infrastructure before they can be considered operational tools for routine crop improvement. An additional perspective is the potential of epigenome editing, which can regulate gene expression in heritable yet reversible and transgene-free soybean.

A key consideration for the future of genome editing in soybean is developing transgene-free or DNA-free edited plants. This can be achieved either through segregation of transgenes by crossing to generate null segregants or by RNP-based editing approaches. While India has adopted a streamlined and comparatively lenient regulatory framework for genome-edited crops and allowed them to bypass the strict biosafety rules for GM crops, this deregulation has prompted significant concerns from activist groups. Targeted sensitization efforts through scientific conferences, public seminars, and transparent stakeholder engagement can help communicate the benefits and safety profile of these techniques to a broader audience. Ultimately, the integration of innovative genome editing platforms, AI-guided experimental design, and supportive policy frameworks will collectively determine the position of soybean as a leading model for next-generation crop improvement and address sustainable production needs across the globe.
